# Synthesis of Heterologous Mevalonic Acid Pathway Enzymes in Clostridium ljungdahlii for the Conversion of Fructose and of Syngas to Mevalonate and Isoprene

**DOI:** 10.1128/AEM.01723-17

**Published:** 2017-12-15

**Authors:** Bruce A. Diner, Janine Fan, Miles C. Scotcher, Derek H. Wells, Gregory M. Whited

**Affiliations:** a Central Research and Development, Biochemical Sciences and Engineering, E. I. du Pont de Nemours and Co., Wilmington, Delaware, USA; b Industrial Biosciences, E. I. du Pont de Nemours and Co., Palo Alto, California, USA; University of Bayreuth

**Keywords:** Clostridium ljungdahlii, acetogenic bacteria, Wood-Ljungdahl pathway, mevalonic acid pathway, syngas, fructose, metabolic engineering, isoprene, mevalonate, bioenergetics

## Abstract

There is a growing interest in the use of microbial fermentation for the generation of high-demand, high-purity chemicals using cheap feedstocks in an environmentally friendly manner. One example explored here is the production of isoprene (C_5_H_8_), a hemiterpene, which is primarily polymerized to polyisoprene in synthetic rubber in tires but which can also be converted to C_10_ and C_15_ biofuels. The strictly anaerobic, acetogenic bacterium Clostridium ljungdahlii, used in all of the work described here, is capable of glycolysis using the Embden-Meyerhof-Parnas pathway and of carbon fixation using the Wood-Ljungdahl pathway. Clostridium-Escherichia coli shuttle plasmids, each bearing either 2 or 3 different heterologous genes of the eukaryotic mevalonic acid (MVA) pathway or eukaryotic isopentenyl pyrophosphate isomerase (Idi) and isoprene synthase (IspS), were constructed and electroporated into C. ljungdahlii. These plasmids, one or two of which were introduced into the host cells, enabled the synthesis of mevalonate and of isoprene from fructose and from syngas (H_2_, CO_2_, and CO) and the conversion of mevalonate to isoprene. All of the heterologous enzymes of the MVA pathway, as well as Idi and IspS, were shown to be synthesized at high levels in C. ljungdahlii, as demonstrated by Western blotting, and were enzymatically active, as demonstrated by *in vivo* product synthesis. The quantities of mevalonate and isoprene produced here are far below what would be required of a commercial production strain. However, proposals are made that could enable a substantial increase in the mass yield of product formation.

**IMPORTANCE** This study demonstrates the ability to synthesize a heterologous metabolic pathway in C. ljungdahlii, an organism capable of metabolizing either simple sugars or syngas or both together (mixotrophy). Syngas, an inexpensive source of carbon and reducing equivalents, is produced as a major component of some industrial waste gas, and it can be generated by gasification of cellulosic biowaste and of municipal solid waste. Its conversion to useful products therefore offers potential cost and environmental benefits. The ability of C. ljungdahlii to grow mixotrophically also enables the recapture, should there be sufficient reducing equivalents available, of the CO_2_ released upon glycolysis, potentially increasing the mass yield of product formation. Isoprene is the simplest of the terpenoids, and so the demonstration of its production is a first step toward the synthesis of higher-value products of the terpenoid pathway.

## INTRODUCTION

Isoprene is a hemiterpene (C_5_H_8_), used primarily for polymerization to *cis*-1,4-polyisoprene, the main constituent of natural rubber (as in vehicle tires and surgical gloves), and for styrene-isoprene-styrene block copolymer (as in thermoplastic rubber and adhesives). Isoprene can also be converted by oligomerization and hydrogenation to produce blendable, saturated C_10_ and C_15_ biofuels (1). The annual global production of petrochemically derived isoprene is close to one million tons (2). With the increase in the volatility of oil prices, clean energy policies likely to result in a decline in U.S. petroleum consumption and thus in the availability of C_5_ fractions from naphtha cracking, and the significant threat of fungal disease to rubber plantations in Asia and Africa (3), there is an increased demand for a more sustainable and reliable generation of isoprene, particularly for the production of synthetic *cis*-1,4-polyisoprene for tires. Isoprene is also the simplest product of the terpenoid pathway. The ability to engineer the production of isoprene is therefore a first step toward the engineering of a host organism to produce this and higher-value terpenoids.

Two evolutionarily distinct pathways exist for the generation of isopentenyl-pyrophosphate (IPP) and dimethylallyl pyrophosphate (DMAPP), the starting point for all terpenoid biosynthesis, including isoprene. These are (i) the 2-*C*-methyl-d-erythritol-4-phosphate/1-deoxy-d-xylulose-5-phosphate (DOXP/MEP) pathway, found in most prokaryotes and which begins with the condensation of pyruvate and glyceraldehyde-3-phosphate, and (ii) the mevalonic acid (MVA) group of pathways found in most eukaryotes, archaea, and some bacteria, which generally begins with the condensation of two acetyl coenzyme A (acetyl-CoA) molecules. Efforts at aerobic microbial biosynthesis of isoprene from glucose, using heterologous protein synthesis of the MVA pathway, have shown higher rates, yields, and titers of isoprene synthesis in Escherichia coli than those from the native DOXP/MEP pathway (2, 4). The exact reason for the higher yields and titers with the MVA pathway is still unclear but may have to do with reduced regulatory control associated with the expression of heterologous MVA pathway genes (e.g., from Saccharomyces cerevisiae and Enterococcus faecalis) or more severe flux bottlenecks in the DOXP/MEP pathway (4, 5).

In the approach demonstrated here, we use fructose and syngas individually as sources of carbon and reducing equivalents (6) to be metabolized to mevalonate and to isoprene in the acetogenic bacterium (7) Clostridium ljungdahlii. This organism is Gram positive and a strict anaerobe, capable of autotrophic growth on H_2_ plus CO_2_ or CO (syngas) or all three together using the Wood-Ljungdahl ([WL] reductive acetyl-CoA) pathway (8, 9), the final product of which is acetyl-CoA. The conversion of syngas to isoprene via acetyl-CoA requires less energy using the MVA pathway than using the DOXP/MEP pathway (which would require gluconeogenesis) and so is the pathway of choice where ATP is limiting. C. ljungdahlii can also grow heterotrophically on organic substrates (e.g., hexoses and pentoses [7, 10]) and mixotrophically, refixing, via the WL pathway, the CO_2_ liberated upon the decarboxylation of pyruvate to acetyl-CoA in glycolysis (7, 11).

A genetic system has been developed for C. ljungdahlii (12, 13), and it has been demonstrated to be capable of heterologous gene expression (12). In this paper, we will show that it is possible to couple the Embden-Meyerhof-Parnas (EMP) and the Wood-Ljungdahl (WL) pathways to a genetically engineered eukaryotic MVA pathway in C. ljungdahlii, enabling the conversions of fructose and syngas to mevalonate and isoprene. Syngas is likely to be a less expensive feedstock than a hexose sugar on a mass basis, as it can be generated by the gasification (via steam or oxygen reforming) of a wide range of inexpensive materials, e.g., natural gas, agricultural waste, coal, and municipal solid waste (6). Low-cost CO-enriched waste streams are also generated as a consequence of industrial processes such as steel manufacture and oil refining, a source that has been exploited by LanzaTech for the generation of biofuels and other biochemicals using acetogenic microorganisms (6). That syngas can be derived from waste gas or from waste materials means that there is potentially an environmental benefit, in addition to a cost benefit, associated with the use of such feedstocks.

## RESULTS

### Plasmids used for the introduction of the MVA pathway genes.

Four different replicons found in Gram-positive organisms are represented in the set of Clostridium-E. coli shuttle plasmids used here (14) (see Materials and Methods). Most of the C. ljungdahlii transformants were obtained with the pCB102 replicon (typically 50 to 100/μg plasmid DNA), approximately one order of magnitude fewer were obtained with pBP1, and none were obtained with pCD6 and pIM13.

### Synthesis of heterologous MvaE and MvaS in pJF100-transformed C. ljungdahlii.

Plasmid pJF100 was constructed (Table 1; see also Fig. S3 and File S2 in the supplemental material) with the heterologous genes *mvaE* and *mvaS* (see Materials and Methods), inserted as an operon under the control of the P*fdx* ferredoxin promoter, with the ferredoxin terminator from C. pasteurianum (*Cpa fdx*). The plasmid was electroporated into C. ljungdahlii (see Materials and Methods and File S2).

**TABLE 1:** Plasmids used and constructed in this work

Plasmid	Gram-positive replicon	Marker	Gram-negative replicon	Promoter and MVA pathway genes
pMTL83151	pCB102	*catP*	ColE1	
pMCS278	pIM13	*ermB*	ColE1	P*fdx*, *mvaE*, *mvaS*
pJF100	pCB102	*catP*	ColE1	P*fdx*, *mvaE*, *mvaS*
pMCS337 (pDW253)	pIM13	*catP*	ColE1	Awo1181, *ispS*, *idi*
pJF100 Fdii	pCB102	*catP*	ColE1	P*fdx*, *ispS*, *idi*
pJF100 Fdii producing N-terminally His-tagged IspS	pCB102	*catP*	ColE1	P*fdx*, N-His-*ispS*, *idi*
pJF100 Fdii producing C-terminally His-tagged IspS	pCB102	*catP*	ColE1	P*fdx*, C-His-*ispS*, *idi*
pJF101	pBP1	*catP*	ColE1	P*thl*, *ispS*, *idi*
pJF102	pCB102	*aad9*	ColE1	P*fdx*, *mvk*, *pmk*, *mvd*
pJF200	pCB102	*catP*	ColE1	Awo1181, *ispS*, *idi*

The *in vivo* synthesis of MvaE and MvaS in pJF100-transformed C. ljungdahlii grown on MES-F medium (chamber gas atmosphere) was compared to that in E. coli TV3007 (Materials and Methods) transformed with the same pJF100 plasmid (15). Western blots (Materials and Methods; see also Fig. S4 and File S2) show high levels of both proteins. The parent bands of MvaE and MvaS showed molecular masses consistent with the calculated 86.5 kDa and 42.1 kDa, respectively (15). However, MvaE was partially proteolyzed, with two fragments running between the 38- and 49-kDa markers.

### Synthesis of mevalonate in C. ljungdahlii grown on fructose.

The *in vivo* enzymatic activity of MvaE and MvaS was demonstrated by the detection of mevalonate in the growth medium. The wild-type (WT) C. ljungdahlii and pMTL83151 (Fig. S3) and pJF100 C. ljungdahlii transformants were grown to an optical density at 600 nm (OD_600_) of ∼2 on MES-F medium in serum bottles, pierced to allow equilibration with the anaerobic chamber atmosphere. Aliquots of the culture medium were acidified to convert mevalonate to mevalonolactone (16) and were spun down, and the supernatants were injected into the high-pressure liquid chromatography (HPLC) instrument (see Materials and Methods and File S2).

**FIG 1:**
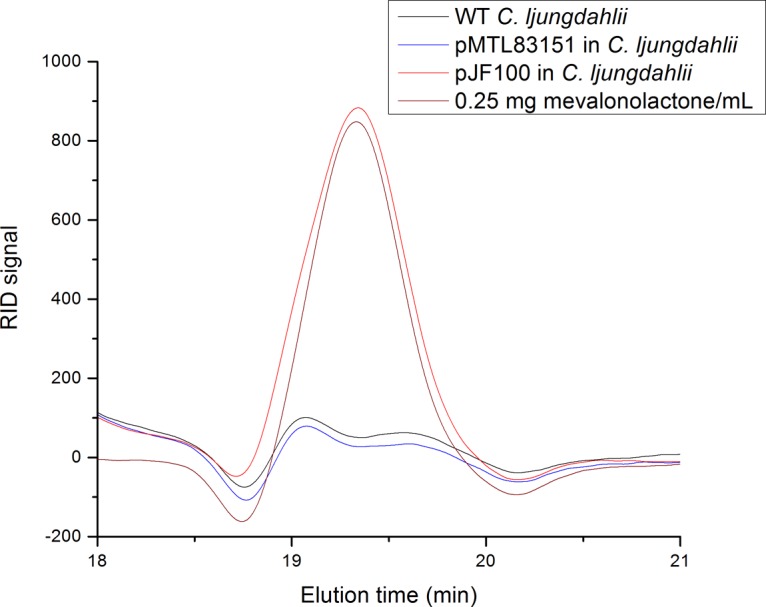
HPLC traces for the detection of mevalonolactone formed by internal esterification of mevalonate, following the acidification of fermentation broth. Samples are C. ljungdahlii WT (black), pMTL83151 transformant (blue), pJF100 transformant (red), and 0.25 mg/ml mevalonolactone (brown). Conditions: 10-μl injection of supernatant of acidified fermentation broth, flow rate of 0.6 ml/min, and detection by a refractive index detector.

The WT and the pMTL83151-transformed C. ljungdahlii showed no peaks in the HPLC elution patterns (Fig. 1) in the position of the mevalonolactone standard (19.3 min, refractive index detector [RID] signal). In contrast, the similarly cultured pJF100 transformant showed a large mevalonolactone signal at this position. Correcting for dilution, there was ∼0.3 mg/ml (2 mM) mevalonate in the culture medium for the pJF100 C. ljungdahlii transformant. Clearly, MvaE and MvaS in the pJF100 plasmid-transformed C. ljungdahlii are active for the conversion of acetyl-CoA to mevalonate.

### Synthesis of heterologous IspS and Idi in pJF100 Fdii-transformed C. ljungdahlii.

**FIG 2:**
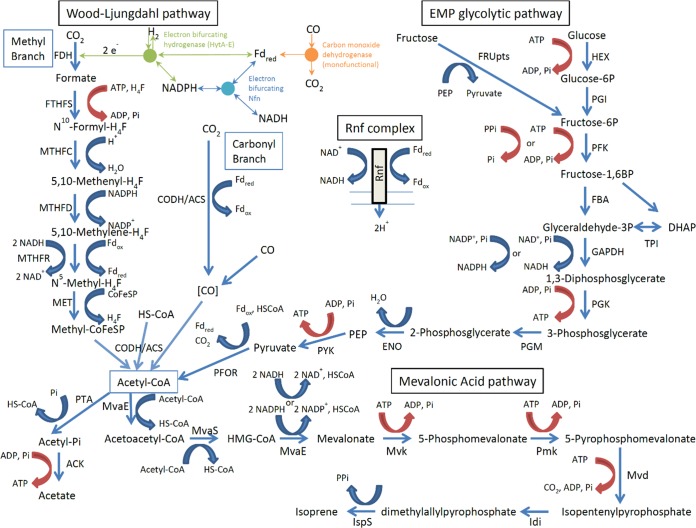
Interactions between the Wood-Ljungdahl pathway, the Embden-Meyerhof-Parnas glycolytic pathway, and the mevalonic acid pathway for the synthesis of isoprene. For the Wood-Ljungdahl pathway: FDH, formate dehydrogenase, which also forms a complex with the electron bifurcating hydrogenase (EBH), HytA-E (21); Nfn, NADH-dependent ferredoxin(red)-NADP oxidoreductase (27); FTHFS, formyltetrahydrofolate synthetase; MTHFC, methenyltetrahydrofolate cyclohydrolase; MTHFD, NADP-dependent methylenetetrahydrofolate dehydrogenase; MTHFR, methylenetetrahydrofolate reductase, putative electron-bifurcating complex (20, 39, but see 25); MET, methyltetrahydrofolate-corrinoid/iron-sulfur protein methyltransferase; CODH/ACS, bifunctional carbon monoxide dehydrogenase/acetyl-CoA synthase; PTA, phosphotransacetylase; ACK, acetate kinase. For the Embden-Meyerhof-Parnas (EMP) glycolytic pathway: FRUpts, fructose phosphoenolpyruvate-dependent phosphotransferase; HEX, hexokinase; PGI, phosphoglucose isomerase; PFK, phosphofructokinase, ATP- and PPi-dependent (36); FBA, fructose bisphosphate aldolase; DHAP, dihydroxyacetone phosphate; TPI, triosephosphate isomerase; GAPDH, glyceraldehyde phosphate dehydrogenase, NAD and NADP-dependent (34); PGK, phosphoglycerate kinase; PGM, phosphoglycerate mutase; ENO, enolase; PEP, phosphoenolpyruvate; PYK, pyruvate kinase; PFOR, pyruvate ferredoxin oxidoreductase. For the mevalonic acid pathway: MvaE, acetyl-CoA acetyltransferase/HMG-CoA reductase, NADPH and NADH dependent (28); MvaS, hydroxymethylglutaryl-CoA synthase; Mvk, mevalonate kinase; Pmk, phosphomevalonate kinase; Mvd, mevalonate diphosphate decarboxylase; Idi, isopentenyl pyrophosphate isomerase; IspS, isoprene synthase; Rnf complex, 6-subunit membrane associated ion-motive complex (19). Reactions involving high-energy phosphate bonds are indicated with red arrows.

The DOXP/MEP pathway, naturally present in C. ljungdahlii (17), generates IPP plus DMAPP. Therefore, the *in vivo* synthesis of Idi and IspS should enable the conversion of endogenous IPP plus DMAPP to isoprene (Fig. 2). Plasmid pJF100 Fdii was constructed (Table 1; see also Fig. S5 and File S2) by replacing the *mvaE* and *mvaS* genes of pJF100 with the heterologous genes *idi* and *ispS* (Materials and Methods). Two preparations of the plasmid (no. 1 and 3) were transferred into C. ljungdahlii.

Western blots of the C. ljungdahlii pJF100 Fdii transformants and of E. coli TV3007, transformed with a related plasmid, pJF200 (Table 1, Fig. S5 and S6, and File S2), show high levels of both proteins. The parent bands of IspS and Idi show molecular masses consistent with the calculated 62.6 kDa and 33.4 kDa, respectively (15), though the IspS was partially proteolyzed, generating two fragments of ∼27 to 28 kDa.

### Conversion of fructose to isoprene.

The *in vivo* enzymatic activity of the translated Idi and IspS was demonstrated by the detection of isoprene in the culture headspace. The C. ljungdahlii WT and the pJF100 Fdii transformants (plasmids 1 and 3) (File S2) were grown for 1 day in 10 ml of MES-F medium in 20-ml Agilent vials at 37°C. The vial headspace was sampled and analyzed by gas chromatography-mass spectrometry ([GC-MS] Materials and Methods). Signals and spectra were compared to those from an isoprene standard.

**FIG 3:**
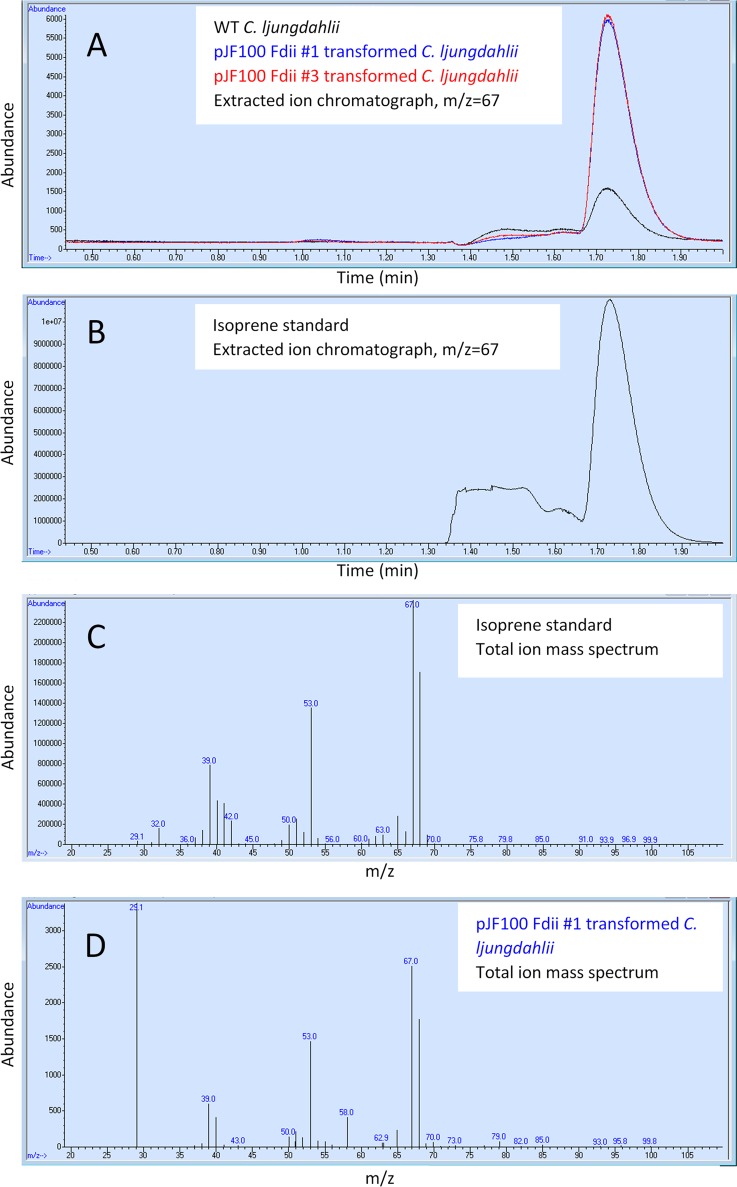
GC-MS detection of isoprene in the culture headspace for C. ljungdahlii cultivated on fructose. C. ljungdahlii WT and pJF100 Fdii transformants were cultivated for 1 day at 37°C on MES-F (see also Table 2 and File S2 in the supplemental material). (A) Gas chromatograms with MS extracted ion detection at an *m/z* of 67 for C. ljungdahlii WT (black) and pJF100 Fdii transformant plasmid 1 (blue) and plasmid 3 (red), following the injection of a 1-ml sample of culture headspace. (B) Gas chromatogram with MS extracted ion detection at an *m/z* of 67 for isoprene standard (1,090 ppm in N_2_) following a 300-μl injection. (C) Total ion MS of isoprene standard (1.70 to 1.80 min) (D) Total ion MS of headspace from culture of C. ljungdahlii pJF100 Fdii plasmid 1 culture (1.70 to 1.80 min) minus the WT spectrum. The OD_600_ values of the cultures at the time of headspace measurement were 1.788, 0.39, and 2.096 for the WT and the pJF100 Fdii transformants with plasmid 1 and plasmid 3, respectively.

The total ion mass spectra (Fig. 3C and D) show a close match between the isoprene standard and the signal derived from the pJF100 Fdii (plasmid 1) transformant headspace, with mass spectrum *m/z* features of 68, 67, 53, and 39. Using single-ion monitoring (*m/z* of 67) (Fig. 3A and B), the C. ljungdahlii WT (black) and the pJF100 Fdii transformants (red and blue) all showed signals with the same retention times as that of the isoprene standard (1.73 min). The WT produced some isoprene, as do other Gram-positive and Gram-negative bacteria (18). However, the transformants generated 4 to 5 times more isoprene (Table 2), indicating that Idi and IspS are enzymatically active in the pJF100 Fdii transformants. The reason for the slower growth of the transformant with plasmid 1 relative to that with plasmid 3 (see Fig. 3 legend) is unclear, and the slower growth was not routinely observed. However, both transformants produced the same amounts of isoprene.

**TABLE 2:** Isoprene determinations for C. ljungdahlii strains

Conditions	Strain	OD_600_	Isoprene amt (ng/ml) in:		Isoprene ratio
			Headspace	Broth	
Fructose to isoprene on MES-F for 1 day at 37°C	WT	1.788	∼1.2	∼1.2	4–5^*a*^
	pFdii transformant plasmid no. 3	2.096	5	5	
Syngas to isoprene on MES-0F for 20 h at 30°C^*b*^	WT	1.034^*d*^	0.077	0.24	6.2^*a*^
	pFdii N-terminally His-tagged IspS transformant	0.888^*d*^	0.48	1.50	
Mevalonate to isoprene on MES-F for 15.5 h at 37°C	pJF101 plus pJF102 no. 4 transformant (no mevalonate added)	1.25^*e*^	∼14	∼9	∼10^*c*^
	pJF101 plus pJF102 no. 4 transformant with 10, 20, and 40 mM mevalonate	No growth^*e*^	140	91	

a Transformant-to-WT ratio.

b First SPME extraction.

c Mevalonate-to-no mevalonate added ratio.

d Initial OD_600_ of 0.500.

e Initial OD_600_ of 0.4.

### C. ljungdahlii adaptation to growth on syngas and 6×His codon *ispS*-modified pJF100 Fdii.

The C. ljungdahlii WT and transformants were adapted to growth on syngas (File S2) to demonstrate that the transformants were capable of producing mevalonate and isoprene from syngas. However, the adaptation of the pJF100 Fdii C. ljungdahlii transformant to growth on MES-0F (syngas atmosphere) required a modification of the plasmid to produce N-terminal and C-terminal 6×His-tagged versions of IspS. Western blotting (Fig. S7) detected Idi and N- and C-terminally His-tagged forms of IspS in the C. ljungdahlii pJF100 Fdii His-tagged IspS transformants, indicating high levels of synthesis for each. These transformants produced isoprene at levels similar to those shown by the pJF100 Fdii transformants when cultivated on fructose (not shown). The reason for the difference in syngas adaptability of the pJF100 Fdii transformants with and without the 6×His tag on IspS is unclear. The doubling times of all of the syngas-adapted strains were typically ≤24 h.

### Conversion of syngas to biomass and product.

To demonstrate that the syngas-adapted C. ljungdahlii pJF100 transformant was indeed converting syngas to cell biomass and product, the cells were cultivated on MES-0F medium plus thiamphenicol at 30°C under either the syngas atmosphere or N_2_ alone. Cultivation experiments were performed in duplicates for 26 h in 4 ml medium in 16.5-ml Supelco vials. Under the syngas atmosphere, the cell density increased from an OD_600_ of 0.500 to 0.822 ± 0.023, while under the N_2_ atmosphere, the cell density increased from an OD_600_ of 0.500 to only 0.520 ± 0. These results indicate that cell growth requires the presence of syngas.

Multiple MES-0F-repassaged syngas cultures of the WT C. ljungdahlii and of the pJF100 and the pJF100 Fdii His-tagged IspS transformants were cultivated on MES-0F under the syngas atmosphere for 26 or 20 h (see Table 3). The final cultures were started at an OD_600_ of 0.500. The cell densities were followed using the OD_600_, the headspaces were analyzed for the percent consumption of CO (∼50%) in that time, and the mole ratio of CO_2_ produced per CO consumed was determined (see Table 3). In all cases, the mole ratio of CO_2_ produced/CO consumed was ≤0.44, indicating that the syngas-adapted C. ljungdahlii pJF100 transformants were fixing CO and not just oxidizing it to CO_2_. These results show that CO was being converted to biomass and to any organic product generated (see below).

**TABLE 3:** Consumption of CO and mole ratio of CO_2_ produced per CO consumed in syngas-cultivated WT and C. ljungdahlii transformants

Syngas-adapted strain	% CO consumed	OD_600_^*a*^	Mole ratio (CO_2_ produced per CO consumed)
pJF100^*b*^	52.65 ± 1.64	0.838	0.237 ± 0.090^*e*^
	51.90 ± 0.14	0.806	0.185 ± 0.010^*d*^
pJF100 Fdii N-terminally His-tagged IspS^*c*^	42	0.888	0.44 ± 0.07^*d*^
pJF100 Fdii C-terminally His-tagged IspS^*c*^	52	0.952	0.34 ± 0.35^*d*^
WT^*c*^	58	1.034	0.41 ± 0.12^*d*^

a Initial OD_600_ of 0.500.

b Grown for 26 h in MES-0F.

c Grown for 20 h in MES-0F.

d Average of two measurements.

e Average of three measurements.

### Production of mevalonate by C. ljungdahlii cultured on syngas.

**FIG 4:**
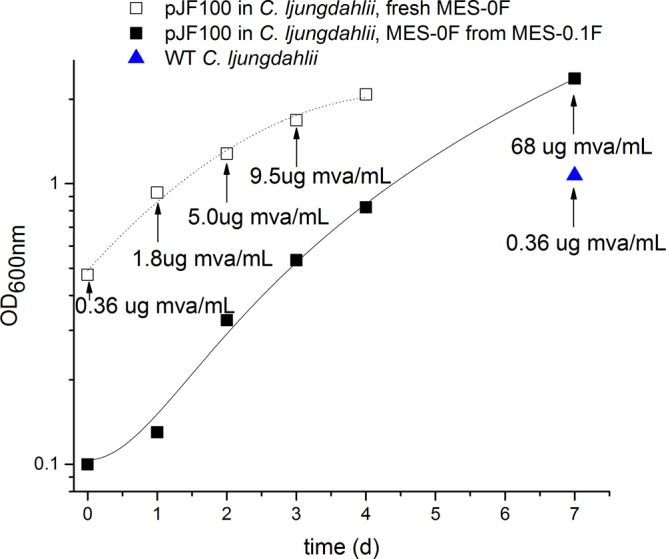
Cultivation on syngas (30°C) and production of mevalonate in syngas-adapted C. ljungdahlii WT and the pJF100 no. 2 transformant. The transformant was initially cultivated in MES-0.1 F with 5 μg thiamphenicol/ml under syngas and diluted 10-fold to an OD_600_ of 0.1 in MES-0F with 5 μg thiamphenicol/ml under syngas. After growing to an OD_600_ of 2.368, the cells were pelleted and resuspended in fresh MES-0F medium with 5 μg thiamphenicol/ml under syngas. Samples were taken on day 7 of the first passage (■) on MES-0F under syngas and on days 1, 2, and 3 of the second passage (□) on MES-0F under syngas. The mevalonate (mva) concentrations were determined by LC-MS. The mevalonate concentration of the syngas-adapted WT C. ljungdahlii (blue triangle, 7-day MES-0F culture under syngas) is equal to that of the MES-0F medium alone. The cell densities were followed by measuring the OD_600_.

The production of mevalonate in the pJF100 C. ljungdahlii transformant cultured on syngas was confirmed using liquid chromatography (LC)-MS (Fig. 4 and Materials and Methods). This strain was subjected to 2 passages, first in MES-0.01F and second in MES-0F (both under a syngas atmosphere), during which time the OD_600_ and the broth mevalonate concentrations were monitored (Fig. 4). The mevalonate concentrations increased with cultivation time (Fig. 4). Syngas-adapted WT C. ljungdahlii, grown on MES-0F (syngas atmosphere) for 7 days to an OD_600_ of 1.07, showed a concentration of mevalonate of 0.36 μg/ml, the same as that present in fresh MES-0F medium. Thus, the WT had produced no mevalonate, in contrast to the pJF100 transformant, which had converted syngas to mevalonate.

### Production of isoprene by C. ljungdahlii grown on syngas.

**FIG 5:**
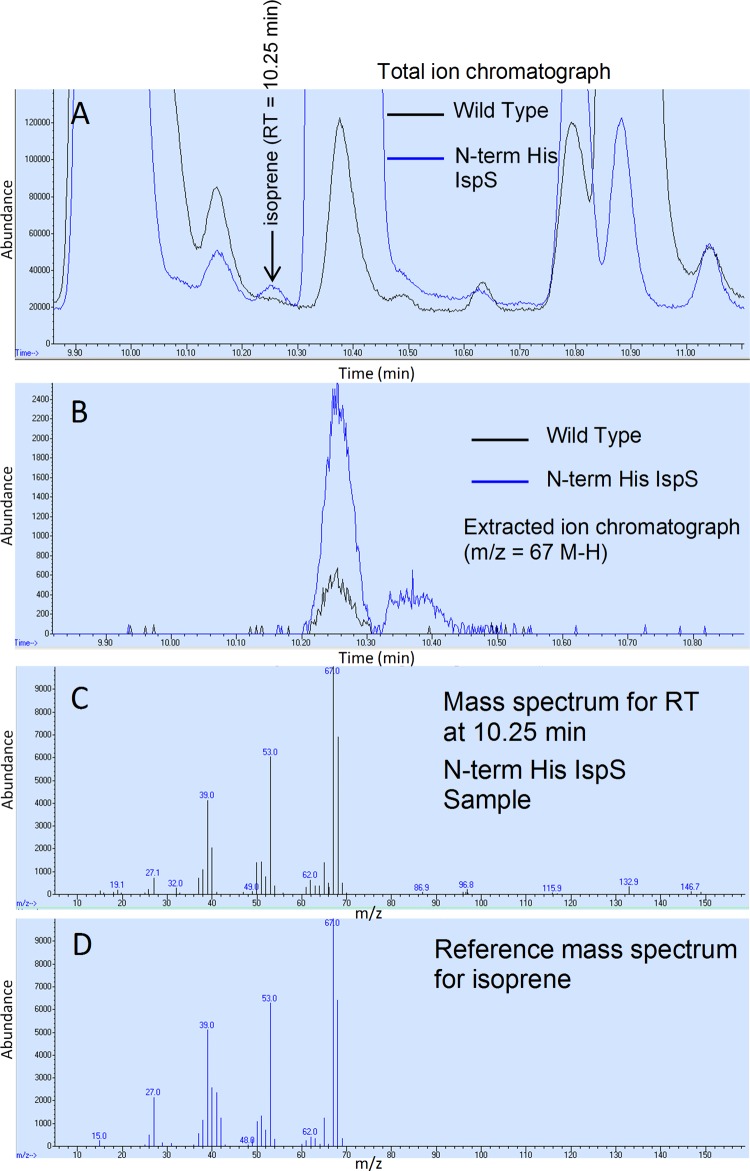
GC-MS detection of isoprene using SPME of culture headspace for C. ljungdahlii cultivated on syngas. C. ljungdahlii WT and the pJF100 Fdii N-terminally His-tagged IspS transformant were cultivated for 20 h at 30°C on MES-0F plus syngas (Table 2 and File S2). After starting at an OD_600_ of 0.5, the final OD_600_ values were 1.034 and 0.888, respectively. The headspace was first sampled for GC determination of CO and CO_2_ (see Table 3) and then separately sampled using SPME for isoprene content (see Table 2). (A) Gas chromatogram of the SPME-desorbed material with MS total ion detection for the WT (black) and the transformant (blue). The isoprene retention time (RT) is 10.25 min as determined by an isoprene standard (not shown). (B) Gas chromatogram with MS detection of isoprene using extracted ion mode (average *m/z* of 67 from 66.70 to 67.70) and splitless injection for the WT (black) and transformant (blue). (C) MS total ion mass spectrum at 10.251 to 10.26 min retention time for the transformant compared to the total ion mass spectrum (D) of an isoprene standard.

The production of isoprene in the pJF100 Fdii N-terminally His-tagged IspS transformant cultured on syngas was confirmed using solid-phase microextraction (SPME) of the headspace coupled with GC-MS (see Materials and Methods). MES-0F-repassaged syngas cultures of the WT C. ljungdahlii and of the pJF100 Fdii N-terminally His-tagged IspS transformant were both started at an OD_600_ of 0.500 in 4 ml MES-0F (syngas atmosphere) in 16.5-ml Supelco vials and grown for 20 h at 30°C (final OD_600_, 1.034 and 0.888, respectively, for the same samples as in Table 3). The total and extracted-ion chromatograms (EICs) for the WT and the pJF100 Fdii N-terminally His-tagged IspS transformant showed elution peaks at 10.25 min (Fig. 5A, B, and C). The mass spectra in each case showed a close fit with the reference isoprene spectrum (Fig. 5D).

Table 2 shows the calculated isoprene concentrations detected on EIC at an *m/z* of 67 at a retention time of 10.25 min. The average from two runs, the first using 20:1 split mode (Table 2) and the second using splitless mode (Fig. 5A, B, C and D) showed that the pJF100 Fdii N-terminally His-tagged IspS strain produced 5.2 ± 1.5 times more isoprene than the C. ljungdahlii WT.

### Synthesis of the lower mevalonate pathway enzymes plus IspS plus Idi.

It remains to be demonstrated that an active lower MVA pathway can be produced in C. ljungdahlii. Plasmid pJF102 (Table 1; see also Fig. S8 and Files S1 and S2) containing the lower pathway genes *mvk*, *pmk*, and *mvd* (see Materials and Methods) was transferred to C. ljungdahlii to enable the production of Mvk, Pmk, and Mvd, respectively (Fig. 2). A second plasmid, pJF101, subsequently transferred (Table 1; see also Fig. S8 and Files S1 and S2), contains *idi* and *ispS*, enabling the conversion to isoprene by Idi and IspS of IPP and DMAPP, produced by the lower MVA pathway. The plasmids contained two different clostridial replicons, antibiotic resistance markers, and promoters (see Table 1) to ensure that both would reside stably within the host cell. Their stepwise transfer into C. ljungdahlii is described in File S2 in the supplemental material.

Two of the doubly transformed C. ljungdahlii strains (pJF101 plus pJF102 no. 4 and 5) were grown on liquid MES-F medium containing both thiamphenicol and spectinomycin. Western blotting (see Fig. S9 and File S2) showed that only no. 4 produced all 5 heterologous proteins.

### Conversion of mevalonate to isoprene.

The *in vivo* conversion of exogenous mevalonate to isoprene was used as a test of enzyme activity. Fresh MES-F media containing 0, 10, 20, and 40 mM mevalonate and both antibiotics were inoculated at an OD_600_ of 0.4 with pJF101 plus pJF102 no.4 double transformants (at 37°C in 10 ml culture medium in 16.5-ml Supelco vials). After 15.5 h, only the vial containing no added mevalonate showed growth (OD_600_ of 1.25). One milliliter of vial headspace was injected into the GC-MS instrument. All three mevalonate-containing cultures produced isoprene (∼140 ng per ml in the headspace, 91 ng isoprene per ml of broth), far higher than the amount of isoprene produced from fructose or syngas (Table 2). The culture with no added mevalonate produced ≤10% of this amount. Clearly, mevalonate was being converted to isoprene in the pJF101 plus pJF102 no. 4 double transformant, indicating that the synthesized heterologous proteins were all enzymatically active.

## DISCUSSION

We have demonstrated that all of the heterologous enzymes of the eukaryotic MVA pathway plus Idi and IspS can be synthesized *in vivo* at high levels and in active forms using Clostridium-E. coli shuttle plasmids in C. ljungdahlii. In transformants cultivated on fructose and on syngas, heterologous MvaE and MvaS (Fig. 2) catalyzed the production of mevalonate from acetyl-CoA, and heterologous Idi and IspS catalyzed the production of isoprene from IPP and DMAPP, produced via the endogenous DOXP/MEP pathway (17). Mvk, Pmk, Mvd, Idi, and IspS (Fig. 2) together were able to catalyze *in vivo* the synthesis of isoprene from exogenous mevalonate.

However, the quantities of product (mevalonate and isoprene) produced here, while a proof of concept, are far below what is required for commercial production. Increased isoprene production is likely to require enhanced levels of heterologous enzyme synthesis and further refinements directed at flux optimization, redox balance, and ATP supply.

### Comparison of cultivation conditions.

The isoprene measurements give some indication of the limitations in pathway flux. Table 2 indicates that considerably more isoprene (18- to 90-fold/cell) accumulates when generated from mevalonate using the lower MVA pathway plus Idi and IspS than when only Idi and IspS are produced in fructose-grown cells. This difference likely results from a higher rate of synthesis and higher intracellular concentrations of IPP and DMAPP produced from mevalonate in the MVA pathway than those produced by the endogenous DOXP/MEP pathway (17) in cells grown on fructose or syngas. An accumulation of toxic phosphorylated intermediates derived from mevalonate, particularly IPP (5), is also a possible explanation for the inhibition of cell growth at ≥10 mM mevalonate. Growth inhibition by ≥10 mM mevalonate itself also remains a possibility. As shown earlier, pJF100-transformed C. ljungdahlii was able to produce 2 mM mevalonate while growing to an OD_600_ of 2 (Fig. 1). Even the level of isoprene produced in the absence of added mevalonate was higher upon the synthesis of the lower mevalonate pathway plus Idi and IspS than in the other cases described in Table 2. MES-F medium contains a very low concentration of mevalonate, 0.36 μg/ml or 2.4 μM (Fig. 4), that is not inhibitory to cell growth and yet is in 20-fold molar excess relative to the amount of isoprene produced (∼1.3 nmol).

Considerably more mevalonate accumulates with fructose as the carbon source than with syngas. With the cells that grew to an OD_600_ of 2 in both cases, ∼300 μg/ml accumulated in the presence of fructose as opposed to ≤68 μg/ml under syngas growth conditions. Growth on syngas is also substantially slower (2- to 4-fold) than it is on fructose. This difference in metabolism is likely a reflection of differences in the cellular energy charge in the two cases, 2 to 3 times higher in fructose-grown as opposed to syngas-grown C. ljungdahlii, as determined in a metabolomics study on C. ljungdahlii (B. A. Diner, Y. Xu, and P. Mauvais, unpublished). Glycolysis produces a net 2 ATP molecules by substrate-level phosphorylation per 2 acetyl-CoA molecules produced in the EMP pathway. Net ATP production from syngas to acetyl-CoA is the result of the operation of the Wood-Ljungdahl pathway (which consumes one ATP/acetyl-CoA molecule) and the chemiosmotic Rnf complex, coupled by the electrochemical membrane potential to the cytoplasmic membrane-bound ATP synthase (19). Assuming that 3.66 H^+^ are required per ATP in the ATP synthase and assuming that all of the electron-bifurcating and Rnf proton pumping mechanisms for energy conservation invoked by Mock et al. (20) (Fig. 2) are operating in C. ljungdahlii in association with the Wood-Ljungdahl pathway, one can calculate that 0.05 ATP are consumed per acetyl-CoA molecule from H_2_ plus CO_2_, and 0.50 ATP per acetyl-CoA are produced from CO. The reason for the difference between H_2_ and CO as the source of reducing equivalents is because CO (E^0′^_CO_2___/CO_ = −520 mV) (21) is more reductive than H_2_ (E^0′^_H^+^__/H_2__ = −414 mV) (22, 23), thereby increasing on a mole basis the amount of reduced ferredoxin (Fd_red_) that can be produced by CO relative to H_2_ and consequently increasing the amount of ATP that can be generated via the proton motive force maintained by the Rnf complex coupled to the reaction Fd_red_ + NAD^+^ → Fd_ox_ + NADH (with a presumed 2H^+^/2e^−^, see references 19 and 20).

The 2 g yeast extract present per liter MES medium had little impact on the flux of carbon to the products in C. ljungdahlii. Syngas-adapted cells cultivated on MES-0F under an N_2_ atmosphere showed no sustained growth. We also found that >2 times as many moles of CO were consumed as moles of CO_2_ produced (Table 3) when C. ljungdahlii was grown on MES-0F and syngas, indicating that a significant fraction of the carbon from CO was converted to biomass and product.

### Bioenergetics considerations for syngas conversion to isoprene.

A total of 6 ATP molecules is required for the synthesis of isoprene from syngas, including 3 for the synthesis of 3 acetyl-CoA molecules via the Wood-Ljungdahl pathway and another 3 for the conversion via the MVA pathway of the 3 acetyl-CoA molecules to isoprene. Bertsch and Müller (24) have argued that in Acetobacterium woodii grown on syngas, an insufficient amount of ATP is generated chemiosmotically to enable the synthesis of isoprene. Some substrate-level ATP production via phosphotransacetylase and acetyl kinase from acetyl-CoA (Fig. 2) is required. The electron-bifurcating redox complexes in C. ljungdahlii differ in numbers and cofactor specificities relative to those in *A. woodii* (20, 24) and offer some bioenergetic advantage over those in *A. woodii*. However, our own calculations (not shown) indicate some shortfall in ATP generation in C. ljungdahlii, even if this organism were to use the same energy-conserving electron-bifurcating complexes suggested to be operating in Clostridium autoethanogenum (20) (Fig. 2), including MTHFR, the presence of which remains speculative (25). As in the redox argument above, it is advantageous in the metabolic pathways to produce NADPH (NADP^+^/NADPH, E′ = −370 mV under physiological conditions) (21) and to consume NADH (NAD^+^/NADH, E′ = −280 mV under physiological conditions) (26), as NADPH (but not NADH) can generate Fd_red_ by Nfn electron bifurcation (Fig. 2) (27). As an example, replacing the NADPH-dependent 3-hydroxy-3-methylglutaryl (HMG)-CoA reductase (MvaE) with one that is NADH dependent (28) adds on a mole basis 1 Fd_red_. In addition, assuming 3.3 H^+^(Na^+^)/ATP (24) or 3.66 H^+^/ATP (20) in the ATP synthase, then the ATP chemiosmotically generated using the Rnf complex is close to the 6 (we calculate 6.2 and 5.7, respectively) molecules necessary for isoprene synthesis from CO. Allowing some substrate-level phosphorylation, derived from acetyl-CoA to acetate (Fig. 2), would provide additional ATP but at a further cost to the mass yield of isoprene synthesis.

There are a number of options, yet to be explored, that might further enhance ATP synthesis. A H^+^-dependent pyrophosphatase has been identified in prokaryotes (29–31), which if introduced into C. ljungdahlii and directed to the cytoplasmic membrane, could contribute (2 H^+^ translocated per PP_i_ hydrolyzed [32]) to the membrane electrochemical proton potential. Doing so would require the use of internal sources of pyrophosphate (e.g., RNA, DNA, polysaccharide, and fatty acyl-CoA synthesis), as an exogenous supply would likely require an expenditure of energy for PP_i_ import.

### Potential for further improvement in the energy balance.

Coelho and coworkers (33) have proposed four anaerobic pathways, beginning with 2,3-dihydroxyisovalerate (DHIV), for the synthesis of isoprene. DHIV can be generated from syngas and the WL pathway by acetyl-CoA → pyruvate → 2-acetolactate → DHIV. While these pathways consume only 1 or 2 ATP molecules as opposed to 3 for the MVA pathway conversion of acetyl-CoA to one isoprene, their realization would require the discovery of 5 to 7 new enzymes.

It was originally shown in Clostridium aceticum and Moorella thermoacetica that the 2 CO_2_ molecules produced per glucose in the EMP pathway could be refixed by the WL pathway using the eight reducing equivalents derived from glycolysis (7). There are sufficient reducing equivalents (24 e^−^) and ATP (six molecules) produced in the glycolysis of 3 fructose molecules to convert the 6 acetyl-CoA molecules generated to 2 isoprenes via the MVA pathway. However, there is then no residual ATP and insufficient reducing equivalents to enable the 6 CO_2_ molecules, generated at the same time, to be refixed and converted to isoprene.

There are potential solutions to both deficiencies. It was recently demonstrated in C. ljungdahlii that H_2_ can provide additional reducing equivalents, enabling, through mixotrophic metabolism, further CO_2_ refixation in the production of acetone (11). A number of pathway modifications of the MVA and glycolytic pathways could enable an enhancement in the generation of ATP as well. These include, as above, the replacement of the NADPH-dependent HMG-CoA reductase with its NADH-dependent homologue in the MVA pathway and, in the EMP pathway, the replacement of the NAD-dependent with an NADP-dependent glyceraldehyde-3-phosphate dehydrogenase (e.g., see reference 34) and replacement of the ATP-dependent phosphofructokinase with its pyrophosphate-dependent homologue (e.g., from Methylomonas methanica [35]) (Fig. 2). The combination of supplemental reducing equivalents, cofactor replacement, and three rather than two ATP molecules per hexose sugar converted to acetyl-CoA via glycolysis (36, 37) could enable, by CO_2_ refixation, the conversion on a mole basis of 3 fructoses to 3 isoprenes. Our calculations (not shown) indicate that these changes in glycolytic substrate-level phosphorylation plus chemiosmotic ATP synthesis could satisfy the overall ATP demand for producing 3 isoprene molecules from 3 fructose molecules with a theoretical mass yield of 37%, an improvement over the 25% yield of the classical EMP and MVA pathways.

## MATERIALS AND METHODS

### Bacterial strains.

Wild-type (WT) Clostridium ljungdahlii (ATCC 55383) and transformants derived therefrom were used for all of the experimental work in this paper. All strains were cultivated anaerobically in MES-F, MES-0.1F, MES-0.01F, or MES-0F (15) medium, containing 10 g/liter, 1 g/liter, 0.1g/liter, or 0 g/liter fructose, respectively, under an atmosphere of anaerobic chamber gas (2% H_2_, 5% CO_2_, and 93% N_2_) or syngas (35% CO, 36% H_2_, 18% CO_2_, and 11% Ar). In MES-0F, syngas was the principal source of carbon and reducing equivalents. The growth of C. ljungdahlii transformants in liquid and solid MES media was routinely carried out in the presence of 5 μg thiamphenicol/ml or 1 mg spectinomycin/ml or both, depending on the plasmid(s) present, unless otherwise indicated.

Genetically engineered E. coli TV3007 was used as a control in Western blotting, as it had previously been shown capable of synthesizing the heterologous enzymes of the MVA pathway. TV3007 is derived from E. coli strain MD12-778 by curing of plasmid pRed-Et. MD12-778 has been described in patent filings US 2013/0273625 and US 2013/0089906.

### Preparation of electrocompetent C. ljungdahlii.

Deoxygenated MES-F medium (15) was inoculated from a frozen stock (MES-F plus 25% glycerol) of WT C. ljungdahlii in a type B anaerobic chamber (Coy Laboratory Products, atmosphere 2% H_2_, 5% CO_2_, and 93% N_2_) and cultured in an incubator shaker (110 rpm; Chemglass Life Sciences) at 37°C. Electrocompetent cells were prepared as described by Leang et al. (13) except that MES-F liquid medium replaced PETC liquid medium. The cells were suspended in a small volume of ice-cold SMP buffer (13) (typically ∼400 μl for 200 ml of initial culture, OD_600_ of 0.1) to which was added a 20% volume of 60% dimethyl sulfoxide (DMSO) and 40% SMP buffer. The suspension was aliquoted in 25-μl volumes into deoxygenated individual cryotubes and quick-frozen using liquid N_2_. The frozen cultures were stored in a liquid N_2_ dewar and showed no loss in electrocompetency over 4 months.

### Plasmid amplification.

Chemically competent E. coli TOP10 cells (Invitrogen) were transformed with plasmid DNA according to the manufacturer's instructions, using 15 μg chloramphenicol/ml or 100 μg spectinomycin/ml as appropriate. Transformants were screened by colony PCR (see Files S1 and S2 in the supplemental material), and correct-sized PCR products were subsequently sequenced. Plasmids were isolated from confirmed transformants using a QIAprep Spin miniprep kit (Qiagen) and eluted with water when used for electroporation into C. ljungdahlii.

### Plasmid components and transformation.

The Clostridium-E. coli shuttle plasmids used for the synthesis of MVA pathway enzymes in C. ljungdahlii were derived from four-part modular shuttle plasmids described by Heap et al. (14). A set of these vectors, pMTL82151, 83151, 83353, 84151, 84422, and 85151, was obtained from the laboratory of Nigel Minton of the University of Nottingham. The genetic elements used were the Gram-negative replicon ColE1 plus tra, the antibiotic resistance markers *catP* (chloramphenicol) and *aad9* (spectinomycin), multiple cloning sites with P*fdx* (from C. sporogenes) and P*thl* (from C. acetobutylicum) promoters and ribosome bindings sites (RBS), and four different Gram-positive replicons, pBP1 *repA* (C. botulinum), pCB102 *repH* (C. butyricum), pCD6 *repA* (C. difficile), and pIM13 *repL* (B. subtilis).

The constructed plasmids (Table 1) bore the genes encoding the MVA pathway enzymes: the upper pathway genes *mvaE* and *mvaS* (both from E. faecalis) (15), encoding MvaE (a fusion of acetyl-CoA acetyltransferase and HMG-CoA reductase) (38) and MvaS (HMG-CoA synthase), respectively; the lower pathway genes *mvk* (from Methanosarcina mazei), *pmk*, and *mvd* (both from S. cerevisiae) (15), encoding MvK (mevalonate kinase), PmK (phosphomevalonate kinase), and Mvd (mevalonate diphosphate decarboxylase), respectively. Also incorporated into the plasmids were *idi* (from S. cerevisiae) and *ispS* (a truncated version from Populus alba) (15), encoding Idi (isopentenyl pyrophosphate isomerase) and IspS (isoprene synthase), respectively.

Plasmid electroporation was similar to that described by Leang et al. (13) with some modification. Twenty-five microliters of electrocompetent C. ljungdahlii cells was thawed on ice in the anaerobic chamber. Two micrograms of plasmid DNA at ∼1 μg/μl was mixed with the cells. The suspension was then placed in a deoxygenated prechilled (on ice) 1-mm gap electroporation cuvette (Bio-Rad) and electroporated (Bio-Rad Gene Pulser Xcell) at 625 V, with a resistance of 600 Ω and a capacitance of 25 μF. Five hundred microliters of deoxygenated, prechilled MES-F medium was added to the cuvette and then transferred to a serum vial containing 10 ml MES-F. The culture was incubated overnight in an incubator shaker (37°C at 110 rpm). The cell suspension was spun at 4,185 × *g* for 15 min, and the pellet was resuspended in 200 μl of MES-F. One hundred microliters was then spread on a 100-mm-diameter petri plate (1.5% agar) containing enriched MES-F medium (MES-F supplemented with 10 g proteose peptone/liter and 10 g beef extract/liter) containing the appropriate antibiotic(s) (5 μg thiamphenicol/ml and/or 1 mg spectinomycin/ml). The agar plates were stored inverted in Oxoid jars (Oxoid Limited), containing anaerobic chamber gas and a palladium catalyst, and placed in an incubator at 37°C. Colonies observed after 3 days were restreaked on enriched and unenriched solid MES-F media containing antibiotic.

### Plasmid recovery from Clostridium ljungdahlii.

The putative transformants were screened by colony PCR using standard conditions with HotStarTaq master mix (Qiagen) and the appropriate primers (Files S1 and S2). Colonies showing the expected-size PCR product were grown in 10 ml liquid MES-F plus antibiotic. Once the cells had reached an OD_600_ of ∼0.2, the culture was spun at 4,185 × *g* (15 min). To each pellet, 250 μl of buffer P1 with RNase A solution (Qiagen) was added along with 50 mg/ml lysozyme (Sigma Life Science). Once resuspended, the cells were removed from the anaerobic chamber and incubated at 37°C for 1 h. After lysis, plasmid DNA was isolated using a QIAprep Spin miniprep kit (Qiagen) according to the kit instructions. Isolated plasmid was used to back-transform chemically competent E. coli TOP10 cells. These were PCR colony screened and sequenced as described under “Plasmid amplification” (above) to verify that the plasmid was fully intact.

### Gel electrophoresis and Western blotting.

In preparation for Western blotting (File S2), frozen phosphate-buffered saline (PBS)-washed cell pellets were thawed and resuspended in ∼1.5 ml PBS (OD_600_ of ∼33) containing 100 μg/ml phenylmethylsulfonyl fluoride (PMSF). The cell suspensions were passaged three times through a French pressure mini-cell (SLM-Aminco) at 138 MPa at ∼5°C. Fifteen microliters of lysate was mixed with 5 μl of NuPAGE LDS sample buffer (Invitrogen) for each lane, and samples were loaded onto Nupage 12% bis-Tris gels (Invitrogen). The gels were run using MES SDS running buffer (Invitrogen) for ∼50 min at constant voltage (200 V).

The proteins were transferred from the gel to a nitrocellulose membrane using an iBlot gel transfer device (Invitrogen). The membrane was then blocked, washed, incubated with primary antibody, washed, incubated with secondary antibody, and washed using blocking solution and wash buffer (Western Breeze; Invitrogen) all per the manufacturer's instructions.

The primary antibody solution was a 1:1,000 (for MvaE, MvaS, Idi, and IspS) and 1:2,000 (for MvK, PmK, and Mvd) dilution of rabbit antiserum in blocking solution (Western Breeze; Invitrogen). The secondary antibody solution was 2 μg/ml Alexa Fluor 488 goat anti-rabbit IgG(H+L) (Life Technologies) in blocking solution. The fluorescent bands were detected in a FluorChem M instrument (ProteinSimple) according to the manufacturer's instructions.

The rabbit polyclonal antisera directed against the MVA pathway enzymes plus Idi and IspS were a gift of Yuliya Primak. The enzymes were His tagged (to aid purification) and synthesized from isopropyl-β-D-thiogalactopyranoside (IPTG)-inducible expression plasmids in E. coli. The antigens were MvaE and MvaS (E. faecalis), Mvk (M. mazei), Pmk, Mvd, and Idi (S. cerevisiae), and IspS (*P. alba*).

### Detection of mevalonolactone by HPLC.

Aliquots of the C. ljungdahlii cultures were acidified to convert mevalonate to mevalonolactone (16) and spun down (14,000 × *g*, 5 min), and the supernatants were injected into a series 1100/1200 HPLC instrument (Agilent Technologies) (File S2). An Aminex HPC-87H ion exclusion column (300 mm by 7.8 mm), fitted with a Micro-guard cation H cartridge guard column (both from Bio-Rad), was equilibrated with a 0.01 N H_2_SO_4_ mobile phase at 60°C at a flow rate of 0.6 ml/min. The sample volumes that were injected were 10 μl. Mevalonolactone was detected by an RID (Agilent), thermostated at 55°C, and eluted between 18 and 21 min. Standard solutions of mevalonolactone in water at 0.25, 1.0, and 4.0 mg/ml were used to calibrate the measurements.

### Detection of mevalonic acid by LC-MS.

C. ljungdahlii cultures were centrifuged (10 min at 4,800 × *g*), and 0.5 ml of supernatant and a 0.1-ml methanol pellet extract were combined and buffered with 10 μl of 1 M ammonium acetate (pH 7.0). Ten-microliter samples were applied to a C_18_ Synergi MAX-RP HPLC column fitted with a manufacturer-recommended guard cartridge (Phenomenex). The column was eluted with a gradient (15) of 15 mM acetic acid plus 10 mM tributylamine in water (solvent A) and LC-MS-grade methanol (solvent B) at a flow rate 0.4 ml/min with a column temperature of 40°C. Mevalonic acid was detected by mass spectrometry using a TSQ quantum triple quadrupole instrument (Thermo Scientific). Mevalonic acid was quantified on the basis of the intensities of the *m/z* 147 peaks, and the 147→59 selected reaction monitoring (SRM) transition was compared to a calibration curve.

### Syngas atmosphere.

A gassing station was used to replace the chamber gas atmosphere (see above) in septa-sealed culture vessels with the syngas atmosphere (35% CO, 36% H_2_, 18% CO_2_, and 11% Ar). The septa were pierced with a hypodermic needle (22 gauge), and the contents of the headspace were evacuated using a vacuum pump to a gauge pressure of −75 kPag. The headspace was then filled with N_2_ to a gauge pressure of 34 kPag. Evacuation and refilling were repeated three times but with syngas instead of N_2_. The final pressure was 34 kPag after the needle was withdrawn.

### Culture headspace detection of CO and CO_2_ using gas chromatography.

Four milliliters of C. ljungdahlii MES-0F cultures was placed in 16.5-ml septa-sealed vials (Supelco) containing syngas and shaken at 30°C overnight. After 20 h, 100 μl was withdrawn from the headspace using a gas-tight syringe (Hamilton) and injected into the splitless injection port of a model 7890B gas chromatograph (Agilent) at 150°C using ultrahigh-purity He as the carrier gas. A CP7429 Select Permanent Gases/CO_2_ column (Varian) was run isothermally at 45°C to separate the components of the sample (CO, H_2_, CO_2_, and Ar), which were detected using a thermal conductivity detector maintained at 200°C.

### GC-MS detection of isoprene in culture headspace using syringe sampling.

One-milliliter samples were taken from the headspace of 10-ml cultures in 16.5-ml Supelco or 20-ml crimp-sealed Agilent vials (as indicated) using a gas-tight syringe (Hamilton). They were injected in splitless mode into the injection port (front inlet temperature, 110°C) of a 7890A gas chromatograph (Agilent) coupled to a 7975C mass spectrometer (Agilent, operating in electron ionization mode). The GC column (HP-5ms; Agilent Technologies) was run at 37°C with He (2 ml/min) as the carrier gas. Extracted ion mass spectra (*m/z* of 67) and total ion mass spectra were compared to those from a 0.3-ml injection of isoprene standard (1,090 ppm isoprene in N_2_).

### GC-MS detection of isoprene in headspace using solid-phase microextraction (SPME).

A 75-μm Carboxen/polydimethylsiloxane (PDMS)-fused silica SPME fiber was used to trap isoprene in the culture vial headspace. The SPME fiber was conditioned by heating at 275°C under helium flow for 15 min. The conditioned fiber was then introduced through the septum into a 16.5-ml Supelco vial containing 4 ml of C. ljungdahlii culture. The fiber was positioned ∼1 cm above the broth and equilibrated at room temperature for 15 min. The fiber was then removed from the vial and immediately inserted into the inlet (at 250°C) of a 7890A/5975C GC-MS instrument (Agilent). The fiber was held in the inlet for 2 min. Desorbed material from the fiber was collected on the chromatographic column (RTX-1, 60 m by 0.320 mm by 3 μm) precooled to −35°C. After desorption, the fiber was removed from the inlet, and the column oven was heated at 20°C per minute to a final temperature of 250°C. The inlet was operated in both split and splitless modes. Calibration was performed using an isoprene standard.

## Supplementary Material

Supplemental material
