# Supplementary material for: Synthesis of Heterologous Mevalonic Acid Pathway Enzymes in Clostridium ljungdahlii for the Conversion of Fructose and of Syngas to Mevalonate and Isoprene
Source: Appl Environ Microbiol. 2017 Dec 15;84(1):e01723-17. doi: 10.1128/AEM.01723-17 (PMC5734045; doi:10.1128/AEM.01723-17)
Supplement: Supplemental material [file supp_84_1_e01723-17__index.html]

Synthesis of Heterologous Mevalonic Acid Pathway Enzymes in Clostridium ljungdahlii for the Conversion of Fructose and of Syngas to Mevalonate and Isoprene — Supplemental material 

# Synthesis of Heterologous Mevalonic Acid Pathway Enzymes in Clostridium ljungdahlii for the Conversion of Fructose and of Syngas to Mevalonate and Isoprene

## Supplemental material

- Supplemental file 1 -

  Nucleotide sequences (File S1); methods (File S2); plasmid maps (Fig. S3, S5 and S8); Western blots (Fig. S4, S6, S7, and S9).

  PDF, 617K
