# Supplementary material for: Synthesis of Heterologous Mevalonic Acid Pathway Enzymes in Clostridium ljungdahlii for the Conversion of Fructose and of Syngas to Mevalonate and Isoprene
Source: Appl Environ Microbiol. 2017 Dec 15;84(1):e01723-17. doi: 10.1128/AEM.01723-17 (PMC5734045; doi:10.1128/AEM.01723-17)
Supplement: Supplemental material [file AEM.01723-17_zam001188217s1.pdf]

SUPPLEMENTARY MATERIAL
Supplementary File S1

pJF100
Upper F4 (SEQ ID NO:13): ggcagtcacg cataacaaag gaatc
Upper R2 (SEQ ID NO:14): ggctgattgg gttcaccgcc atttg
UpperF1 (SEQ ID NO:15): aaaagacatt ccactatttc tgaag
UpperR6 (SEQ ID NO:16): aaacatttcc aaagattact tgatc
CatP-F (SEQ ID NO:17): aggtttaaac ttagggtaac aaaaaacacc gtatttctac

pJF200
pMCS337IspSFOR (SEQ ID NO:30): atggaagcaa gaagaagtgc
pMCS337IspSFREV (SEQ ID NO:31): cctctagtcc ttataacacc tatctv
pMCS337IDIFor (SEQ ID NO:32): aggtgttata aggactagag gaaaatgagg
PCB102F (SEQ ID NO:33): gcttgtagct aagtagtacg aaagg
PCB102R (SEQ ID NO:34): atccttttgt atcggctcac tacac

pJF100 Fdii
vec100_fwd (Seq ID No. 36): gtcaaaaggc ataacagtgc tgaatag
vec100_rev (Seq ID No. 37): atgtaacaca cctccttaaa aattacacaa c
II_insert200_fwd (Seq ID No. 38): taatttttaa ggaggtgtgt tacatatgga agcaagaaga agtgcaaact acgaa
II_insert200_rev (Seq ID No. 39): attcagcact gttatgcctt ttgactatca c
Fdx For1 (Seq ID No. 40): gatgtagata ggataataga atcc
UP mcs Mint (Seq ID No. 41): atcaggaaac agctatgacc gc
Isp seq F1 (Seq ID No. 42): gatttgaaag tgatataaga ggtg gatttgaaag tgatataaga ggtg
Isp seq F2 (Seq ID No. 43): gaacttgaac tttttacaga tgc gaacttgaac tttttacaga tgc
Isp seq F3 (Seq ID No. 44): tgaatcttat agatgaaaca tgg
Isp seq F4 (Seq ID No. 45): agaacacaaa gacttgaagc ag
Isp seq R1 (Seq ID No. 46): caggacttgt atgtgcatca ccat
Isp seq R2 (Seq ID No. 47): gatcatttat tgcatttaca tccc
Isp seq R3 (Seq ID No. 48): tgaagagatg tttttgttac tgc
Id seq F1 (Seq ID No. 49): gacagcagac aataattcta tgcc
Id seq F2 (Seq ID No. 50): taaggagaat ctaacagtaa accc
Id seq F3 (Seq ID No. 51): gaaaattaga tcacgagctt ggc
Id seq R1 (Seq ID No. 52): cacttaaatc atctagctgc tccc
Id seq R2 (Seq ID No. 53: ctctttgttg taataaaagt tcgcc

repH seq F1 (Seq ID No. 54): tgtacgttct tttttctgtt cttcc

pJF100 Fd ii His-tagged Isps

N-Forward (SEQ ID NO:56): ttaaggaggt gtgttacata tgcatcacca tcaccatcac gaagcaagaa gaagtgcaaa c

N-Reverse (SEQ ID NO:57): gtttgcactt cttcttgctt cgtgatggtg atggtgatgc atatgtaaca cacctcctta a

C-Forward (SEQ ID NO:58): cctatacttc cttttgaaag acatcaccat caccatcact aggtgttata aggactagag g

C-Reverse (SEQ ID NO:59): cctctagtcc ttataacacc tagtgatggt gatggtgatg tctttcaaaa ggaagtatag g

FdxF1 (SEQ ID NO:60): gatgtagata ggataataga atcc

IDIR2 (SEQ ID NO:61): ctctttgttg taataaaagt tcgcc

The SEQ ID NO:XX are also found in ref. [1].

pJF102 (P*fdx* mmp pCB102 *aad9*)

For+XmaI: CATCCCGGGA GGAGGTTAGT TCATATGGTG TCA

Rev+NheI: CATGCTAGCT ATTCAGCACT GTTATGTTAT TTATCT

For-mvk F2:GGTGATACAG GAGTTTTCAG CAGCAC

Rev-mvdR: GCCATACTAT CATGTCCATC CTCAGC

pJF101 (P*thl* ii pBP1 *catP*)

pBP1For2: GTAATCTGCT GCTTGCAAAC AAAAAAAC

pBP1Rev2: CCACAAACTA TTAAAGTTAA ACATAAAAAT AACATCG

pMCS337IspSFOR: ATGGAAGCAA GAAGAAGTGC

pMCS337IspSFREV:CCTCTAGTCC TTATAACACC TATCT

pMCS337IDIFor: AGGTGTTATA AGGACTAGAG GAAAATGAGG

Pmcs337IDIR2: TTAAAGCATT CTATGTATTT GCCTATCATT TTC

Supplementary File S1

Nucleotide sequence of plasmid pJF101 (Table 1) (6939 bp)

```
                                                                                                        NotI
                                                                                                      ~~~~~~~~
   1      CCTGCAGGAT AAAAAAATTG TAGATAAATT TTATAAAATA GTTTTATCTA CAATTTTTTT ATCAGGAAAC AGCTATGACC GCGGCCGCTT TTTAACAAAA
 101      TATATTGATA AAAATAATAA TAGTGGGTAT AATTAAGTTG TTAGAGAAAA CGTATAAATT AGGGATAAAC TATGGAACTT ATGAAATAGA TTGAAATGGT
                                                                          NdeI
                                                                         ~~~~~~
 201      TTATCTGTTA CCCCGTATCA AAATTTAGGA GGTTAGTTCA TTAATTTTTA AGGAGGTGTG TTACATATGG AAGCAAGAAG AAGTGCAAAC TACGAACCTA
 301      ATAGTTGGGA TTATGATTAT CTTCTTAGTA GTGATACAGA TGAAAGTATA GAAGTATATA AAGATAAAGC AAAAAAACTT GAAGCAGAAG TAAGAAGAGA
 401      AATAAATAAC GAAAAAGCAG AATTTCTTAC ACTTCTTGAA CTTATAGATA ATGTACAAAG ACTTGGACTT GGATATAGAT TTGAAAGTGA TATAAGAGGT
 501      GCACTTGATA GATTTGTAAG TAGTGGAGGA TTTGATGCAG TAACAAAAAC ATCTCTTCAT GGAACAGCTC TTAGTTTTAG ACTTCTTAGA CAACATGGAT
 601      TTGAAGTAAG TCAAGAAGCA TTTTCTGGAT TTAAAGATCA AAATGGAAAT TTTCTTGAAA ATCTTAAAGA AGATATAAAA GCAATACTTA GTCTTTATGA
 701      AGCAAGTTTT CTTGCACTTG AAGGTGAAAA TATACTTGAT GAAGCAAAAG TATTTGCAAT AAGTCATCTT AAAGAACTTA GTGAAGAAAA AATAGGAAAA
 801      GAACTTGCAG AACAAGTAAA TCATGCACTT GAACTTCCAC TTCATAGAAG AACACAAAGA CTTGAAGCAG TATGGTCTAT AGAAGCATAT AGAAAAAAAG
 901      AAGATGCAAA TCAAGTACTT CTTGAACTTG CAATACTTGA TTATAATATG ATACAAAGTG TATATCAAAG AGATCTTAGA GAAACAAGTA GATGGTGGAG
1001      AAGAGTAGGA CTTGCAACAA AACTTCATTT TGCAAGAGAT AGACTTATAG AAAGTTTTTA TTGGGCAGTT GGAGTAGCAT TTGAACCTCA ATATAGTGAT
1101      TGTAGAAATA GTGTAGCAAA AATGTTTAGT TTTGTAACAA TAATAGATGA TATATACGAT GTATATGGAA CACTTGATGA ACTTGAACTT TTTACAGATG
                                                                                                                NdeI
                                                                                                               ~~~~~~
1201      CAGTAGAAAG ATGGGATGTA AATGCAATAA ATGATCTTCC TGATTATATG AAACTTTGTT TTCTTGCTCT TTATAATACA ATAAATGAAA TAGCATATGA
1301      TAATCTTAAA GATAAAGGTG AAAATATTCT TCCTTATCTT ACAAAAGCAT GGGCAGATCT TTGTAATGCA TTTCTTCAAG AGGCAAAATG GCTTTATAAT
1401      AAAAGTACAC CTACATTTGA TGATTATTTT GGAAATGCAT GGAAAAGTAG TAGTGGACCT CTTCAACTTG TATTTGCATA TTTTGCAGTA GTACAAAATA
1501      TTAAAAAAGA AGAAATAGAA AATCTTCAAA AATATCATGA TACAATAAGT AGACCTAGTC ATATATTTAG ACTTTGTAAT GATCTTGCAA GTGCAAGTGC
1601      AGAAATAGCA AGAGGTGAAA CAGCAAATAG TGTAAGTTGT TATATGAGAA CAAAAGGTAT AAGTGAAGAA CTTGCAACAG AAAGTGTAAT GAATCTTATA
1701      GATGAAACAT GGAAAAAAAT GAATAAAGAA AAACTTGGAG GAAGTCTTTT TGCAAAACCT TTTGTAGAAA CAGCAATAAA TCTTGCAAGA CAAAGTCATT
1801      GTACATATCA TAATGGTGAT GCACATACAA GTCCTGATGA GCTTACAAGA AAAAGAGTAC TTTCTGTAAT AACAGAACCT ATACTTCCTT TTGAAAGATA
                                                                                                     NdeI
                                                                                                    ~~~~~~
1901      GGTGTTATAA GGACTAGAGG AAAATGAGGA GTTGTTATGA ATGACAGCAG ACAATAATTC TATGCCACAT GGAGCAGTAT CTTCATATGC TAAATTAGTT
2001      CAGAACCAAA CCCCTGAAGA CATTTTAGAA GAATTTCCTG AAATTATACC ATTACAGCAA AGACCTAATA CAAGAAGTTC TGAAACATCA AATGATGAAT
2101      CAGGTGAAAC TTGCTTTTCT GGACATGACG AAGAACAGAT TAAATTAATG AATGAAAATT GCATTGTACT TGATTGGGAT GATAATGCTA TAGGAGCTGG
2201      TACTAAAAAG GTGTGTCATT TAATGGAAAA CATAGAGAAG GGTTTACTTC ATAGAGCATT CTCAGTTTTC ATATTTAATG AACAGGGCGA ACTTTTATTA
2301      CAACAAAGAG CAACAGAGAA AATTACTTTC CCTGATTTAT GGACAAATAC TTGTTGCAGT CACCCATTAT GCATAGATGA TGAGTTGGGT TTGAAAGGTA
2401      AATTAGATGA TAAAATAAAA GGTGCTATAA CCGCTGCAGT TAGAAAATTA GATCACGAGC TTGGCATACC AGAAGATGAG ACTAAAACAA GAGGTAAATT
2501      TCACTTCTTG AATAGGATTC ACTATATGGC TCCTAGTAAT GAGCCTTGGG GTGAACACGA AATTGATTAC ATACTATTTT ATAAAATAAA CGCTAAGGAG
2601      AATCTAACAG TAAACCCAAA TGTAAATGAG GTTAGAGACT TTAAGTGGGT TAGCCCAAAT GATCTTAAAA CTATGTTTGC AGATCCATCA TATAAGTTTA
2701      CACCTTGGTT TAAAATAATT TGCGAAAACT ACCTTTTTAA TTGGTGGGAG CAGCTAGATG ATTTAAGTGA AGTAGAAAAT GATAGGCAAA TACATAGAAT
2801      GCTTTAAGCT AGCATAAAAA TAAGAAGCCT GCATTTGCAG GCTTCTTATT TTTATGGCGC GCCGTTCTGA ATCCTTAGCT AATGGTTCAA CAGGTAACTA
2901      TGACGAAGAT AGCACCCTGG ATAAGTCTGT AATGGATTCT AAGGCATTTA ATGAAGACGT GTATATAAAA TGTGCTAATG AAAAAGAAAA TGCGTTAAAA
3001      GAGCCTAAAA TGAGTTCAAA TGGTTTTGAA ATTGATTGGT AGTTTAATTT AATATATTTT TTCTATTGGC TATCTCGATA CCTATAGAAT CTTCTGTTCA
3101      CTTTTGTTTT TGAAATATAA AAAGGGGCTT TTTAGCCCCT TTTTTTTAAA ACTCCGGAGG AGTTTCTTCA TTCTTGATAC TATACGTAAC TATTTTCGAT
3201      TTGACTTCAT TGTCAATTAA GCTAGTAAAA TCAATGGTTA AAAAACAAAA AACTTGCATT TTTCTACCTA GTAATTTATA ATTTTAAGTG TCGAGTTTAA
3301      AAGTATAATT TACCAGGAAA GGAGCAAGTT TTTTAATAAG GAAAAATTTT TCCTTTTAAA ATTCTATTTC GTTATATGAC TAATTATAAT CAAAAAAATG
3401      AAAATAAACA AGAGGTAAAA ACTGCTTTAG AGAAATGTAC TGATAAAAAA AGAAAAAATC CTAGATTTAC GTCATACATA GCACCTTTAA CTACTAAGAA
3501      AAATATTGAA AGGACTTCCA CTTGTGGAGA TTATTTGTTT ATGTTGAGTG ATGCAGACTT AGAACATTTT AAATTACATA AAGGTAATTT TTGCGGTAAT
3601      AGATTTTGTC CAATGTGTAG TTGGCGACTT GCTTGTAAGG ATAGTTTAGA AATATCTATT CTTATGGAGC ATTTAAGAAA AGAAGAAAAT AAAGAGTTTA
3701      TATTTTTAAC TCTTACAACT CCAAATGTAA AAAGTTATGA TCTTAATTAT TCTATTAAAC AATATAATAA ATCTTTTAAA AAATTAATGG AGCGTAAGGA
3801      AGTTAAGGAT ATAACTAAAG GTTATATAAG AAAATTAGAA GTAACTTACC AAAAGGAAAA ATACATAACA AAGGATTTAT GGAAAATAAA AAAAGATTAT
3901      TATCAAAAAA AAGGACTTGA AATTGGTGAT TTAGAACCTA ATTTTGATAC TTATAATCCT CATTTTCATG TAGTTATTGC AGTTAATAAA AGTTATTTTA
4001      CAGATAAAAA TTATTATATA AATCGAGAAA GATGGTTGGA ATTATGGAAG TTTGCTACTA AGGATGATTC TATAACTCAA GTTGATGTTA GAAAAGCAAA
4101      AATTAATGAT TATAAAGAGG TTTACGAACT TGCGAAATAT TCAGCTAAAG ACACTGATTA TTTAATATCG AGGCCAGTAT TTGAAATTTT TTATAAAGCA
4201      TTAAAAGGCA AGCAGGTATT AGTTTTTAGT GGATTTTTTA AAGATGCACA CAAATTGTAC AAGCAAGGAA AACTTGATGT TTATAAAAAG AAAGATGAAA
4301      TTAAATATGT CTATATAGTT TATTATAATT GGTGCAAAAA ACAATATGAA AAAACTAGAA TAAGGGAACT TACGGAAGAT GAAAAAGAAG AATTAAATCA
4401      AGATTTAATA GATGAAATAG AAATAGATTA AAGTGTAACT ATACTTTATA TATATATGAT TAAAAAAATA AAAAACAACA GCCTATTAGG TTGTTGTTTT
4501      TTATTTTCTT TATTAATTTT TTTAATTTTT AGTTTTTAGT TCTTTTTTAA AATAAGTTTC AGCCTCTTTT TCAATATTTT TTAAAGAAGG AGTATTTGCA
4601      TGAATTGCCT TTTTTCTAAC AGACTTAGGA AATATTTTAA CAGTATCTTC TTGCGCCGGT GATTTTGGAA CTTCATAACT TACTAATTTA TAATTATTAT
4701      TTTCTTTTTT AATTGTAACA GTTGCAAAAG AAGCTGAACC TGTTCCTTCA ACTAGTTTAT CATCTTCAAT ATAATATTCT TGACCTATAT AGTATAAATA
4801      TATTTTTATT ATATTTTTAC TTTTTTCTGA ATCTATTATT TTATAATCAT AAAAAGTTTT ACCACCAAAA GAAGGTTGTA CTCCTTCTGG TCCAACATAT
4901      TTTTTTACTA TATTATCTAA ATAATTTTTG GGAACTGGTG TTGTAATTTG ATTAATCGAA CAACCAGTTA TACTTAAAGG AATTATAACT ATAAAAATAT
5001      ATAGGATTAT CTTTTTAAAT TTCATTATTG GCCTCCTTTT TATTAAATTT ATGTTACCAT AAAAAGGACA TAACGGGAAT ATGTAGAATA TTTTTAATGT
5101      AGACAAAATT TTACATAAAT ATAAAGAAAG GAAGTGTTTG TTTAAATTTT ATAGCAAACT ATCAAAAAATT AGGGGGATAA AAATTTATGA AAAAAAGGTT
5201      TTCGATGTTA TTTTTATGTT TAACTTTAAT AGTTTGTGGT TTATTTACAA ATTCGGCCGG CCAGTGGGCA AGTTGAAAAA TTCACAAAAA TGTGGTATAA
5301      TATCTTTGTT CATTAGAGCG ATAAACTTGA ATTTGAGAGG GAACTTAGAT GGTATTTGAA AAAATTGATA AAAATAGTTG GAACAGAAAA GAGTATTTTG
5401      ACCACTACTT TGCAAGTGTA CCTTGTACCT ACAGCATGAC CGTTAAAGTG GATATCACAC AAATAAAGGA AAAGGGAATG AAACTATATC CTGCAATGCT
5501      TTATTATATT GCAATGATTG TAAACCGCCA TTCAGAGTTT AGGACGGCAA TCAATCAAGA TGGTGAATTG GGGATATATG ATGAGATGAT ACCAAGCTAT
5601      ACAATATTTC ACAATGATAC TGAAACATTT TCCAGCCTTT GGACTGAGTG TAAGTCTGAC TTTAAATCAT TTTTAGCAGA TTATGAAAGT GATACGCAAC
5701      GGTATGGAAA CAATCATAGA ATGGAAGGAA AGCCAAATGC TCCGGAAAAC ATTTTTAATG TATCTATGAT ACCGTGGTCA ACCTTCGATG GCTTTAATCT
5801      GAATTTGCAG AAAGGATATG ATTATTTGAT TCCTATTTTT ACTATGGGGA AATATTATAA AGAAGATAAC AAAATTATAC TTCCTTTGGC AATTCAAGTT
5901      CATCACGCAG TATGTGACGG ATTTCACATT TGCCGTTTTG TAAACGAATT GCAGGAATTG ATAAATAGTT AACTTCAGGT TTGTCTGTAA CTAAAAACAA
6001      GTATTTAAGC AAAAACATCG TAGAAATACG GTGTTTTTTG TTACCCTAAG TTTAAACTCC TTTTTGATAA TCTCATGACC AAAATCCCTT AACGTGAGTT
6101      TTCGTTCCAC TGAGCGTCAG ACCCCGTAGA AAAGATCAAA GGATCTTCTT GAGATCCTTT TTTTCTGCGC GTAATCTGCT GCTTGCAAAC AAAAAAACCA
6201      CCGCTACCAG CGGTGGTTTG TTTGCCGGAT CAAGAGCTAC CAACTCTTTT TCCGAAGGTA ACTGGCTTCA GCAGAGCGCA GATACCAAAT ACTGTTCTTC
6301      TAGTGTAGCC GTAGTTAGGC CACCACTTCA AGAACTCTGT AGCACCGCCT ACATACCTCG CTCTGCTAAT CCTGTTACCA GTGGCTGCTG CCAGTGGCGA
6401      TAAGTCGTGT CTTACCGGGT TGGACTCAAG ACGATAGTTA CCGGATAAGG CGCAGCGGTC GGGCTGAACG GGGGGTTCGT GCACACAGCC CAGCTTGGAG
6501      CGAACGACCT ACACCGAACT GAGATACCTA CAGCGTGAGC TATGAGAAAG CGCCACGCTT CCCGAAGGGA GAAAGGCGGA CAGGTATCCG GTAAGCGGCA
6601      GGGTCGGAAC AGGAGAGCGC ACGAGGGAGC TTCCAGGGGG AAACGCCTGG TATCTTTATA GTCCTGTCGG GTTTCGCCAC CTCTGACTTG AGCGTCGATT
6701      TTTGTGATGC TCGTCAGGGG GGCGGAGCCT ATGGAAAAAC GCCAGCAACG CGGCCTTTTT ACGGTTCCTG GCCTTTTGCT GGCCTTTTGC TCACATGTTC
6801      TTTCCTGCGT TATCCCCTGA TTCTGTGGAT AACCGTATTA CCGCCTTTGA GTGAGCTGAT ACCGCTCGCC GCAGCCGAAC GACCGAGCGC AGCGAGTCAG
6901      TGAGCGAGGA AGCGGAAGAG CGCCCAATAC GCAGGGCCC
```

Nucleotide sequence of plasmid pJF102 (Table 1) (8284 bp)

```
   1  CCTGCAGGAT AAAAAAATTG TAGATAAATT TTATAAAATA GTTTTATCTA CAATTTTTTT ATCAGGAAAC AGCTATGACC GCGGCCGCGT GTAGTAGCCT
 101  GTGAAATAAG TAAGGAAAAA AAAGAAGTAA GTGTTATATA TGATGATTAT TTTGTAGATG TAGATAGGAT AATAGAATCC ATAGAAAATA TAGGTTATAC
 201  AGTTATATAA AAATTACTTT AAAAATTAAT AAAAACATGG TAAAATATAA ATCGTATAAA GTTGTGTAAT TTTTAAGGAG GTGTGTTACA TATGACCATG
                                   XmaI
                                  ~~~~~~~
 301  ATTACGAATT CGAGCTCGGT ACATCCCGGG CAGGAGGTTA GTTCATATGG TGTCATGTAG TGCACCAGGA AAAATTTACT TATTTGGAGA ACATGCAGTG
 401  GTGTACGGTG AAACAGCAAT TGCTTGCGCA GTAGAACTTA GAACAAGGGT AAGGGCAGAG TTAAATGATA GTATAACCAT ACAGTCTCAA ATAGGAAGAA
 501  CCGGACTAGA TTTCGAAAAA CATCCTTATG TTTCAGCTGT TATAGAGAAG ATGAGGAAGA GCATACCAAT AAATGGCGTT TTCTTAACAG TAGACTCTGA
 601  CATTCCTGTA GGTAGCGGTT TGGGAAGTTC AGCAGCAGTT ACTATTGCAA GTATAGGAGC TTTAAACGAG CTTTTTGGAT TTGGCTTGTC ATTGCAAGAG
 701  ATTGCTAAGC TTGGACACGA AATAGAAATT AAAGTACAAG GAGCTGCAAG TCCTACTGAT ACTTATGTAT CAACATTTGG TGGAGTAGTT ACTATACCAG
 801  AAAGAAGAAA GTTAAAAACT CCAGATTGTG GAATAGTGAT AGGTGATACA GGAGTTTTCA GCAGCACCAA AGAGCTAGTT GCTAACGTTA GACAATTAAG
 901  GGAATCTTAT CCTGACTTAA TTGAACCTCT TATGACTAGC ATAGGCAAAA TATCAAGAAT AGGAGAGCAA TTGGTTTTAA GCGGAGACTA TGCTTCAATT
1001  GGCAGGTTGA TGAATGTGAA TCAGGGTTTA TTAGACGCTT TGGGAGTAAA CATACTTGAG TTATCTCAGT TAATATATAG TGCAAGGGCA GCTGGAGCAT
1101  TTGGAGCAAA GATAACAGGT GCTGGTGGTG GTGGATGTAT GGTAGCATTA ACAGCTCCTG AAAAATGCAA TCAAGTTGCA GAAGCTGTAG CAGGAGCAGG
1201  TGGTAAGGTT ACTATAACCA AACCTACTGA ACAAGGACTT AAGGTAGACT AGGCTAGTGT ATCAAAATTT AGGAGGTTAG TTCATATGAC AGTTTACACT
1301  GCTTCTGTAA CAGCACCAGT TAACATAGCT ACATTAAAAT ATTGGGGAAA AAGGGATACA AAGTTGAATT TGCCAACAAA TTCAAGCATA TCTGTTACAC
1401  TTAGCCAAGA TGACCTTAGA ACCTTAACTT CTGCAGCAAC TGCACCAGAA TTCGAAAGAG ATACATTATG GTTAAATGGT GAGCCTCATT CAATAGATAA
1501  TGAAAGAACA CAAAATTGCT TAAGAGACTT AAGACAGTTA AGAAAGGAAA TGGAGTCTAA AGATGCAAGC TTACCTACTC TTAGTCAGTG GAAATTACAC
1601  ATTGTGAGCG AAAACAATTT TCCTACTGCA GCTGGTCTTG CATCATCAGC TGCTGGTTTT GCTGCTCTTG TAAGCGCTAT TGCAAAGTTA TACCAATTAC
1701  CTCAAAGTAC ATCTGAAATA AGTAGAATAG CAAGAAAAGG TAGTGGAAGT GCTTGTAGAT CATTATTTGG TGGATATGTT GCTTGGGAAA TGGGAAAAGC
1801  TGAGGATGGA CATGATAGTA TGGCTGTTCA GATAGCAGAT TCATCAGATT GGCCACAGAT GAAAGCTTGT GTATTAGTTG TTAGCGATAT AAAGAAGGAT
1901  GTATCAAGTA CACAAGGAAT GCAGTTGACT GTGGCAACAA GCGAACTTTT TAAAGAGAGG ATAGAGCACG TTGTACCAAA AAGATTTGAA GTTATGAGGA
2001  AAGCAATAGT AGAAAAGGAT TTCGCTACTT TCGCAAAGGA AACTATGATG GATTCTAACA GTTTTCATGC AACATGCCTT GACAGCTTCC CACCAATATT
2101  CTACATGAAT GATACTTCAA AAAGGATAAT AAGTTGGTGT CACACTATAA ACCAATTTTA TGGTGAGACA ATTGTTGCTT ATACATTTGA TGCAGGACCA
2201  AATGCTGTTT TATATTATCT AGCAGAAAAC GAGTCTAAGT TGTTTGCATT CATATATAAA CTTTTCGGAT CTGTACCAGG ATGGGATAAG AAATTTACTA
2301  CTGAGCAGTT GGAAGCATTC AACCATCAAT TTGAGTCAAG TAACTTTACT GCTAGAGAGC TTGACCTTGA ATTACAAAAG GACGTTGCAA GGGTTATTCT
2401  TACTCAGGTA GGTAGTGGTC CACAGGAAAC CAACGAGTCA TTGATAGATG CAAAAACTGG ATTACCTAAA GAATAAGCTA GTGTATCAAA ATTTAGGAGG
2501  TTAGTTCATA TGAGTGAACT TAGAGCATTT TCTGCTCCTG GAAAAGCATT ATTAGCAGGA GGCTACTTAG TTCTTGATAC AAAATATGAA GCATTTGTGG
2601  TGGGTTTATC AGCTAGAATG CATGCAGTTG CTCATCCTTA TGGTTCTCTT CAAGGATCAG ATAAATTTGA AGTAAGAGTG AAATCTAAAC AGTTTAAAGA
2701  TGGTGAATGG CTATACCACA TAAGCCCTAA AAGTGGCTTT ATTCCTGTAT CAATAGGAGG CTCAAAGAAT CCTTTTATTG AGAAGGTAAT AGCAAACGTA
2801  TTCTCATACT TTAAACCAAA TATGGATGAT TATTGCAACA GAAATCTTTT TGTAATTGAT ATATTTTCAG ATGATGCTTA TCATTCTCAA GAAGATAGCG
2901  TAACAGAACA CAGAGGAAAT AGAAGACTAA GTTTTCACAG CCATAGAATT GAAGAAGTAC CTAAAACTGG ACTTGGTTCT TCAGCAGGAT TAGTTACCGT
3001  ATTAACTACT GCATTGGCAA GCTTCTTTGT TTCTGATTTA GAAAACAACG TGGATAAATA TAGAGAAGTA ATACACAACT TAGCTCAAGT AGCTCATTGT
3101  CAGGCTCAAG GCAAGATAGG CTCTGGATTT GATGTTGCTG CTGCTGCTTA TGGATCTATT AGATATAGAA GATTTCCACC AGCTTTAATA TCAAATCTTC
3201  CAGATATAGG ATCAGCAACA TACGGTTCTA AATTAGCTCA TCTTGTAGAT GAAGAGGATT GGAACATAAC TATAAAGAGC AACCATTTAC CTTCAGGCTT
3301  GACACTATGG ATGGGTGATA TTAAAAATGG ATCAGAGACA GTTAAGTTAG TTCAGAAAGT TAAAAACTGG TATGATTCAC ATATGCCTGA AAGTTTAAAG
3401  ATATACACTG AATTGGACCA CGCAAACAGC AGATTTATGG ATGGTTTGAG CAAGTTGGAT AGATTACATG AAACTCACGA TGATTATTCA GATCAGATTT
3501  TTGAATCTCT TGAAAGAAAT GATTGCACAT GCCAGAAGTA TCCAGAAATA ACAGAGGTTA GGGATGCAGT TGCTACAATT AGAAGAAGCT TTAGAAAAAT
3601  TACTAAAGAG TCAGGCGCAG ACATAGAACC TCCTGTTCAA ACAAGTTTGC TTGATGATTG TCAAACACTT AAAGGAGTAC TTACTTGTTT AATACCTGGT
3701  GCAGGAGGAT ATGATGCTAT AGCAGTAATA ACAAAGCAAG ATGTAGATTT AAGAGCACAG ACAGCAAACG ATAAAAGATT CTCTAAAGTG CAGTGGTTAG
                                                                                                       NheI
                                                                                                      ~~~~~~
3801  ATGTTACTCA GGCTGATTGG GGAGTAAGAA AAGAGAAGGA TCCAGAAACT TACCTAGATA AATAACATAA CAGTGCTGAA TAGCTAGCAT GAGACTAGCA
3901  TAAAAATAAG AAGCCTGCAT TTGCAGGCTT CTTATTTTTA TGGCGCGCCG CCATTATTTT TTTGAACAAT TGACAATTCA TTTCTTATTT TTTATTAAGT
4001  GATAGTCAAA AGGCATAACA GTGCTGAATA GAAAGAAATT TACAGAAAAG AAAATTATAG AATTTAGTAT GATTAATTAT ACTCATTTAT GAATGTTTAA
4101  TTGAATACAA AAAAAAATAC TTGTTATGTA TTCAATTACG GGTTAAAATA TAGACAAGTT GAAAAATTTA ATAAAAAAAT AAGTCCTCAG CTCTTATATA
4201  TTAAGCTACC AACTTAGTAT ATAAGCCAAA ACTTAAATGT GCTACCAACA CATCAAGCCG TTAGAGAACT CTATCTATAG CAATATTTCA AATGTACCGA
4301  CATACAAGAG AAACATTAAC TATATATATT CAATTTATGA GATTATCTTA ACAGATATAA ATGTAAATTG CAATAAGTAA GATTTAGAAG TTTATAGCCT
4401  TTGTGTATTG GAAGCAGTAC GCAAAGGCTT TTTTATTTGA TAAAAATTAG AAGTATATTT ATTTTTTCAT AATTAATTTA TGAAAATGAA AGGGGGTGAG
4501  CAAAGTGACA GAGGAAAGCA GTATCTTATC AAATAACAAG GTATTAGCAA TATCATTATT GACTTTAGCA GTAAACATTA TGACTTTTAT AGTGCTTGTA
4601  GCTAAGTAGT ACGAAAGGGG GAGCTTTAAA AAGCTCCTTG GAATACATAG AATTCATAAA TTAATTTATG AAAAGAAGGG CGTATATGAA AACTTGTAAA
4701  AATTGCAAAG AGTTTATTAA AGATACTGAA ATATGCAAAA TACATTCGTT GATGATTCAT GATAAAACAG TAGCAACCTA TTGCAGTAAA TACAATGAGT
4801  CAAGATGTTT ACATAAAGGG AAAGTCCAAT GTATTAATTG TTCAAAGATG AACCGATATG GATGGTGTGC CATAAAAATG AGATGTTTTA CAGAGGAAGA
4901  ACAGAAAAAA GAACGTACAT GCATTAAATA TTATGCAAGG AGCTTTAAAA AAGCTCATGT AAAGAAGAGT AAAAAGAAAA AATAATTTAT TTATTAATTT
5001  AATATTGAGA GTGCCGACAC AGTATGCACT AAAAAATATA TCTGTGGTGT AGTGAGCCGA TACAAAAGGA TAGTCACTCG CATTTTCATA ATACATCTTA
5101  TGTTATGATT ATGTGTCGGT GGGACTTCAC GACGAAAACC CACAATAAAA AAAGAGTTCG GGGTAGGGTT AAGCATAGTT GAGGCAACTA AACAATCAAG
5201  CTAGGATATG CAGTAGCAGA CCGTAAGGTC GTTGTTTAGG TGTGTTGTAA TACATACGCT ATTAAGATGT AAAAATACGG ATACCAATGA AGGGAAAAGT
5301  ATAATTTTTG GATGTAGTTT GTTTGTTCAT CTATGGGCAA ACTACGTCCA AAGCCGTTTC CAAATCTGCT AAAAAGTATA TCCTTTCTAA AATCAAAGTC
5401  AAGTATGAAA TCATAAATAA AGTTTAATTT TGAAGTTATT ATGATATTAT GTTTTTCTAT TAAAATAAAT TAAGTATATA GAATAGTTTA ATAATAGTAT
5501  ATACTTAATG TGATAAGTGT CTGACAGTGT CACAGAAAGG ATGATTGTTA TGGATTATAA GCGGCCGGCC CAATGAATAG GTTTACACTT ACTTTAGTTT
5601  TATGGAAATG AAAGATCATA TCATATATAA TCTAGAATAA AATTAACTAA AATAATTATT ATCTAGATAA AAAATTTAGA AGCCAATGAA ATCTATAAAT
5701  AAACTAAATT AAGTTTATTT AATTAACAAC TATGGATATA AAATAGGTAC TAATCAAAAT AGTGAGGAGG ATATATTTGA ATACATACGA ACAAATTAAT
5801  AAAGTGAAAA AAATACTTCG GAAACATTTA AAAAATAACC TTATTGGTAC TTACATGTTT GGATCAGGAG TTGAGAGTGG ACTAAAACCA AATAGTGATC
5901  TTGACTTTTT AGTCGTCGTA TCTGAACCAT TGACAGATCA AAGTAAAGAA ATACTTATAC AAAAAATTAG ACCTATTTCA AAGAAAATAG GAGATAAAAG
6001  CAACTTACGA TATATTGAAT TAACAATTAT TATTCAGCAA GAAATGGTAC CGTGGAATCA TCCTCCCAAA CAAGAATTTA TTTATGGAGA ATGGTTACAA
6101  GAGCTTTATG AACAAGGATA CATTCCTCAG AAGGAATTAA ATTCAGATTT AACCATAATG CTTTACCAAG CAAAACGAAA AAATAAAAGA ATATACGGAA
6201  ATTATGACTT AGAGGAATTA CTACCTGATA TTCCATTTTC TGATGTGAGA AGAGCCATTA TGGATTCGTC AGAGGAATTA ATAGATAATT ATCAGGATGA
6301  TGAAACCAAC TCTATATTAA CTTTATGCCG TATGATTTTA ACTATGGACA CGGGTAAAAT CATACCAAAA GATATTGCGG GAAATGCAGT GGCTGAATCT
6401  TCTCCATTAG AACATAGGGA GAGAATTTTG TTAGCAGTTC GTAGTTATCT TGGAGAGAAT ATTGAATGGA CTAATGAAAA TGTAAATTTA ACTATAAACT
6501  ATTTAAATAA CAGATTAAAA AAATTATAAA AAAATTGAAA AAATGGTGGA AACACTTTTT TCAATTTTTT TGTTTTATTA TTTAATATTT GGGAAATATT
6601  CATTCTAATT GGTAATCAGA TTTTAGAAGT TTAAACTCCT TTTTGATAAT CTCATGACCA AAATCCCTTA ACGTGAGTTT TCGTTCCACT GAGCGTCAGA
6701  CCCCGTAGAA AAGATCAAAG GATCTTCTTG AGATCCTTTT TTTCTGCGCG TAATCTGCTG CTTGCAAACA AAAAAACCAC CGCTACCAGC GGTGGTTTGT
6801  TTGCCGGATC AAGAGCTACC AACTCTTTTT CCGAAGGTAA CTGGCTTCAG CAGAGCGCAG ATACCAAATA CTGTTCTTCT AGTGTAGCCG TAGTTAGGCC
6901  ACCACTTCAA GAACTCTGTA GCACCGCCTA CATACCTCGC TCTGCTAATC CTGTTACCAG TGGCTGCTGC CAGTGGCGAT AAGTCGTGTC TTACCGGGTT
7001  GGACTCAAGA CGATAGTTAC CGGATAAGGC GCAGCGGTCG GGCTGAACGG GGGGTTCGTG CACACAGCCC AGCTTGGAGC GAACGACCTA CACCGAACTG
7101  AGATACCTAC AGCGTGAGCT ATGAGAAAGC GCCACGCTTC CCGAAGGGAG AAAGGCGGAC AGGTATCCGG TAAGCGGCAG GGTCGGAACA GGAGAGCGCA
7201  CGAGGGAGCT TCCAGGGGGA AACGCCTGGT ATCTTTATAG TCCTGTCGGG TTTCGCCACC TCTGACTTGA GCGTCGATTT TTGTGATGCT CGTCAGGGGG
7301  GCGGAGCCTA TGGAAAAACG CCAGCAACGC GGCCTTTTTA CGGTTCCTGG CCTTTTGCTG GCCTTTTGCT CACATGTTCT TTCCTGCGTT ATCCCCTGAT
7401  TCTGTGGATA ACCGTATTAC CGCCTTTGAG TGAGCTGATA CCGCTCGCCG CAGCCGAACG ACCGAGCGCA GCGAGTCAGT GAGCGAGGAA GCGGAAGAGC
7501  GCCCAATACG CAGGGCCCCC TGCTTCGGGG TCATTATAGC GATTTTTTCG GTATATCCAT CCTTTTTCGC ACGATATACA GGATTTTGCC AAAGGGTTCG
7601  TGTAGACTTT CCTTGGTGTA TCCAACGGCG TCAGCCGGGC AGGATAGGTG AAGTAGGCCC ACCCGCGAGC GGGTGTTCCT TCTTCACTGT CCCTTATTCG
7701  CACCTGGCGG TGCTCAACGG GAATCCTGCT CTGCGAGGCT GGCCGGCTAC CGCCGGCGTA ACAGATGAGG GCAAGCGGAT GGCTGATGAA ACCAAGCCAA
7801  CCAGGAAGGG CAGCCCACCT ATCAAGGTGT ACTGCCTTCC AGACGAACGA AGAGCGATTG AGGAAAAGGC GGCGGCGGCC GGCATGAGCC TGTCGGCCTA
7901  CCTGCTGGCC GTCGGCCAGG GCTACAAAAT CACGGGCGTC GTGGACTATG AGCACGTCCG CGAGCTGGCC CGCATCAATG GCGACCTGGG CCGCCTGGGC
8001  GGCCTGCTGA AACTCTGGCT CACCGACGAC CCGCGCACGG CGCGGTTCGG TGATGCCACG ATCCTCGCCC TGCTGGCGAA GATCGAAGAG AAGCAGGACG
8101  AGCTTGGCAA GGTCATGATG GGCGTGGTCC GCCCGAGGGC AGAGCCATGA CTTTTTTAGC CGCTAAAACG GCCGGGGGGT GCGCGTGATT GCCAAGCACG
8201  TCCCCATGCG CTCCATCAAG AAGAGCGACT TCGCGGAGCT GGTGAAGTAC ATCACCGACG AGCAAGGCAA GACCGATCGG GCCC
```

Supplementary File S2

Strain Construction and Western Blotting

Construction of plasmid pJF100 (Table 1) for *in vivo* synthesis of heterologous MvaE and MvaS

The *mvaE* (DNA SEQ ID NO:7; protein SEQ ID NO:8) (1) and *mvaS* coding regions (DNA SEQ ID NO:9; protein SEQ ID NO:10) (1) both from *Enterococcus faecalis* were cloned as an operon under the control of the P*fdx* ferredoxin promoter (from *C. sporogenes*), with the *C. pasteurianum* ferredoxin terminator (Cpa *fdx* terminator), in a modular vector with a pIM13 Gram positive replicon, the ColE1 origin of replication for *E. coli*, and the *ermB* marker creating pMCS278 (DNA SEQ ID NO:23) (2) (Supplementary Figure S3). The vector pMTL83151(3), also named pMCS201 (DNA SEQ ID NO:24) in Beck et al. (2), carries the pCB102 Gram positive origin of replication, the *catP* marker, and the ColE1 *E. coli* origin of replication (Supplementary Figure S3).

The vector pMCS278 was digested with restriction enzymes, *Pme*I and *Asc*I (New England Biolabs, Inc.), and the 4.8 kb fragment gel purified (QIAquick Gel Extraction Kit (Qiagen Inc), removing the *ermB* marker and Gram positive replicon pIM13. Plasmid pMTL83151 was digested with *Pme*I, *Asc*I and *Apa*I and gel purified, yielding a 2.4 kb insert containing the *catP* marker and the pCB102 replicon. The vector fragment and insert were ligated with T4 DNA ligase (New England Biolabs Inc) at room temperature overnight and the product transformed into chemically-competent *E. coli* Top10 cells (Invitrogen). After outgrowth in S.O.C. medium (Invitrogen), aliquots of the transformation mix were plated onto Lysogeny Broth (LB) plates with 15 µg chloramphenicol/mL. The plates were incubated overnight at 30°C.

Transformants were screened by colony PCR with HotStarTaq Master Mix (Qiagen) using primers (see Supplementary File S1) Upper F4 (SEQ ID NO:13) and Upper R2 (SEQ ID NO:14) (1). Several colonies that PCR-amplified with the correct-sized product (640 bp) were

sequenced by TempliPhi (GE Health Care Life Sciences) using primers (see Supplementary File S1) UpperF1 (SEQ ID NO:15), UpperR6 (SEQ ID NO:16), UpperF4 (SEQ ID NO:13), UpperR2 (SEQ ID NO:14), and CatP-F (SEQ ID NO:17) (1). Confirmed transformants were grown in LB medium (30°C) with chloramphenicol selection (15 µg/mL), pelleted (5000 × g, 10 min) and the plasmids isolated from the pellet using QIAprep Spin Miniprep Kit (Qiagen). The final plasmid elution was performed in water to enable electroporation into *C. ljungdahlii*. The resulting plasmid was named pJF100 (SEQ ID NO:18) (1) (see Supplementary Figure S3).

Plasmid pJF100 was electroporated into electrocompetent *C. ljungdahlii* (see Materials and Methods). Sixty colonies of putative transformants were obtained after 3 days following anaerobic growth at 37°C on enriched MES-F plates (see Materials and Methods) containing 5 µg thiamphenicol/mL. Eight of these were restreaked onto MES-F agar plates containing 5 µg thiamphenicol/mL and grown up in an Oxoid jar at 37°C for several days. Four of these were screened by colony PCR to verify transformation and grown anaerobically on liquid MES-F medium + 5 µg thiamphenicol/mL at 37°C. These cultures were then used for mini plasmid preps (see Materials and Methods). The isolated plasmids were then back-transformed into electrocompetent *E. coli* Top10. Plasmids isolated from the back-transformants were verified for intactness by DNA sequencing as described above. Confirmed *C. ljungdahlii* transformants were grown anerobically in liquid MES-F + 5 µg thiamphenicol/mL at 37°C, pelleted, resuspended in an equal volume of 50% glycerol and frozen in liquid N_2.

Western blotting to detect expression of MvaE and MvaS

C. ljungdahlii WT and pJF100 transformants #2, #4, #6 and #8 were grown anerobically from frozen stocks in 200 mL MES-F liquid medium + 5 µg thiamphenicol/mL at 37°C. At

harvest, the cell densities (OD_{600}) were 0.864, 0.912, 0.634, 0.854 and 0.882, respectively. The cell suspensions were poured into sealed 50 mL Falcon tubes, removed from the anaerobic chamber and spun at 5000 rpm (4800 × g) for 10' at 5°C in a HS-4 Sorvall rotor. The tubes were returned to the anaerobic chamber and the pellets were resuspended and spun down as before in PBS (Phosphate buffered saline – 137 mM NaCl, 2.7 mM KCl, 10 mM Na_2HPO_4, 2.0 mM KH_2PO_4, pH 7.4). The final washed pellets were frozen in liquid N_2 and stored in a -80°C freezer.

In preparation for Western blotting, the pellets were thawed and resuspended in 1.5 mL PBS containing 100 µg/mL phenylmethylsulfonyl fluoride (PMSF). The cell suspensions were passaged three times through a French pressure Mini-Cell (SLM-Aminco) at 138 MPa at ~5°C. A portion of the lysates was used immediately for Western blotting and the remainder aliquoted out, quick frozen in liquid N_2 and stored in a -80°C freezer. Fifteen µL of lysate was mixed with 5 µL of LDS sample buffer (Invitrogen) for each lane of each of three gels – two for Western blots and one Simple Blue SafeStain (Thermo Fisher Scientific)-stained gel. SDS-PAGE gel electrophoresis and Western blotting were carried out as described in Materials and Methods (Supplementary Figure S4).

Production of mevalonate in pJF100-transformed *C. ljungdahlii* grown on fructose

Crimp-capped bottles (160 mL total internal volume) containing 10 mL of MES-F medium were inoculated from MES-F agar plates of WT *C. ljungdahlii*. Similar crimp-capped bottles with 10 mL of MES-F medium + 5 µg thiamphenicol/mL were inoculated with cells of pMTL83151- and with pJF100-transformed *C. ljungdahlii* from enriched MES-F plates + 5 µg thiamphenicol/mL. The culture bottles were sealed with septa and crimp caps inside the

anaerobic chamber. The septa of the 160 mL bottles were pierced with a 22-gauge sterile needle and capped by a sterile Super Acrodisc 13 (0.2 μm, Gelman Sciences), allowing the atmosphere within the culture bottles to equilibrate with the chamber gas (2% H_2, 5% CO_2 and 93% N_2). All vials and bottles were placed on an incubator shaker at 37°C and 110 rpm (Incu-Shaker Mini Shaking Incubator; Chemglass Life Sciences) inside the anaerobic chamber. The cultures were allowed to grow until they reached OD_{600} of ~2. The OD_{600} at harvesting for these cultures were 2.04 for WT, 1.904 for the pMTL83151 transformant and 2.064 for the pJF100 transformant. At this point 300 μL of culture was placed in 1.5 mL Eppendorf Flex-Tubes (Eppendorf North America) to which were added 54 μL each of 10% H_2SO_4 to convert mevalonate to mevalonolactone for HPLC determination of mevalonate (4). The samples were mixed and incubated for ≥45 min at 4°C. The samples were spun in an Eppendorf centrifuge at 14,000 × g for 5 min to pellet the cells, at which point the supernatants were loaded into HPLC vials with 200 μL inserts for injection into the HPLC (see Materials and Methods and Figure 2).

Construction of pJF200 (Table 1)

A vector (pJF200) for the expression of IspS (isoprene synthase) and Idi (isopentenyl diphosphate isomerase) using the *Acetobacterium woodii* promoter, Awo1181gi, with a pCB102 Gram positive replicon, was derived from the plasmids pMTL83151 (Supplementary Figure S3) and pMCS337 (also called pDW253; SEQ ID NO:23(1); plasmid map Supplementary Figure S5). Further details on the construction of plasmid pJF200 are provided in Beck et al. (1).

C. ljungdahlii was transformed by electroporation with pJF200 as previously described for pJF100. The transformants obtained, carrying pJF200, showed no evidence, however, of IspS or Idi expression by Western blotting (not shown). Plasmid isolated from these strains, used to

back transform *E. coli* TV3007, did, however, express IspS and Idi (by Western blot, Supplementary Figure S6) indicating that in the proper host the pJF200 plasmid was functional for expression. We concluded that while active in *E. coli* TV3007, the *A. woodii* promoter Awo1181gi was not active in *C. ljungdahlii* under the growth conditions used. Consequently, this promoter was replaced with the *C. sporogenes* P*fdx* promoter that was used for MvaE and MvaS expression from plasmid pJF100.

Construction of plasmid pJF100 Fdii (Table 1)

Genes *ispS* and *idi* were placed under the control of the P*fdx* promoter by replacing the *mva*E and *mva*S in vector pJF100 with these coding regions. A 3.5-kb DNA vector fragment which contains the P*fdx* promoter was PCR amplified using pJF100 as template and primers (see Supplementary File S1) vec100_fwd (Seq ID No. 36) and vec100_rev (Seq ID No. 37) (1). The resulting PCR product was digested with *Dpn* I (New England Biolabs, Inc.) for 30 min at 37°C to remove methylated template DNA and heat inactivated at 80°C for 20 min. The ~3.5-kb PCR product was gel purified using a Zymoclean™ Gel DNA Recovery Kit (Zymo Research Corporation).

A 2.7 kb DNA fragment containing *ispS* and *idi* was PCR amplified using pJF200 as template and primers (see Supplementary File S1) II_insert200_fwd (Seq ID No. 38) and II_insert200_rev (Seq ID No. 39) (1) using Q5® High-Fidelity 2X Master Mix (New England Biolabs, Inc.). The 2.7 kb PCR product was digested with *Dpn* I and gel purified as in the preceding paragraph.

The ~2.7-kb *ispS*/*idi* insert fragment and the ~3.5-kb vector fragment were combined, using 100 ng of the vector DNA and at least 2-fold excess of the insert, and assembled using a

Gibson Assembly® Cloning Kit (New England Biolabs). After incubation for 15 min at 50°C, a chilled aliquot of the reaction mix was used to transform High Efficiency NEB 5-alpha Competent *E. coli* (New England Biolabs Inc.). After a 2-hour outgrowth in S.O.C. medium at 30°C, aliquots of the transformation mix were spread onto LB plates containing 15 μg chloramphenicol/mL and incubated overnight at 30° C. Transformants were screened through colony PCR screening using primers (see Supplementary File S1) Fdx For1 (Seq ID No. 40) and Isp seq R2 (Seq ID No. 47) (1). Plasmid DNA was prepared from cell pellets of successful transformants using a QIAprep Spin Miniprep Kit (Qiagen) and sequenced using primers (see Supplementary File S1) UP mcs Mint (Seq ID No. 41), Isp seq F1 (Seq ID No. 42), Isp seq F2 (Seq ID No. 43), Isp seq F3 (Seq ID No. 44), Isp seq F4 (Seq ID No. 45), Isp seq R1 (Seq ID No. 46), Isp seq R2 (Seq ID No. 47), Isp seq R3 (Seq ID No. 48), Id seq F1 (Seq ID No. 49), Id seq F2 (Seq ID No. 50), Id seq F3 (Seq ID No. 51), Id seq R1 (Seq ID No. 52), Id seq R2 (Seq ID No. 53), and repH seq F1 (Seq ID No. 54) (1). The resulting ~ 6.2-kb plasmid was named pJF100 Fdii (Supplementary Figure S5) (Seq ID No. 55) (1).

Electrocompetent *C. ljungdahlii* was transformed with two different preparations of pJF100 Fdii plasmid, called plasmid 1 and plasmid 3, isolated from Top 10 cells as described earlier. Four colonies of putative transformants, two from each of the two plasmid preps were picked from the enriched MES-F + 5 μg thiamphenicol/mL plates and were restreaked on MES-F + 5 μg thiamphenicol/mL plates. These were used to inoculate 50 mL cultures of MES-F + 5 μg thiamphenicol/mL. Cultures of pJF100 Fdii #1, plasmid 1, #4 plasmid 1, #9 plasmid 3 and #11 plasmid 3 were grown anaerobically at 37°C to an OD_{600} of 0.724, 0.768, 0.610 and 0.670. The cell suspensions were harvested and washed with PBS as described earlier for Western blotting

of the pJF100 *C. ljungdahlii* transformants. The cells were pelleted, frozen in liquid N_2 and stored in a -80°C freezer.

In preparation for Western blotting, the pellets were thawed and resuspended in 1.4, 1.5, 1.2 and 1.3 mL, respectively, of PBS containing 100 µg/mL PMSF. The different volumes were used to assure approximately equal cell densities. The cell suspensions were passaged three times through a French pressure Mini-Cell (SLM-Aminco) at 138 MPa at ~5°C. A portion of the lysates were used immediately for Western blotting and the remainder aliquoted out, quick frozen in liquid N_2 and stored in a -80°C freezer. Fifteen µL of lysate was mixed with 5 µL of LDS sample buffer (Invitrogen) for each lane of each of three gels: two for Western blots and one stained with Simple Blue SafeStain.

Transformants of *E. coli* TV3007 were prepared as described earlier (1) with plasmid isolated from two *C. ljungdahlii* pJF200 Fdii transformants named nos.1LD and 2LD. IspS and Idi were synthesized using the Awo1181 promoter in *E. coli* TV3007 and were used as markers for IspS and Idi. These transformants were grown up as described earlier (see also Beck et al. (1)) and harvested and solubilized with LDS sample buffer as before.

Gel electrophoresis and Western blotting were performed as described in the Materials and Methods section (see Supplementary Figure S6).

Adaptation of *C. ljungdahlii* to growth on syngas

Adaptation to growth on syngas was carried out by culturing *C. ljungdahlii* cells in liquid medium at decreasing concentrations of fructose from MES-F to MES-0.1F to MES-0F medium under a syngas atmosphere (35% CO, 36% H_2, 18% CO_2 and 11% Ar), after which the cells were cultured on MES-0F plates under syngas and colonies selected. Adaptation was successfully

carried out for *C. ljungdahlii* WT and the pJF100 transformant. Adaptation of the pJF100 Fdii *C. ljungdahlii* transformant to growth on MES-0F under a syngas atmosphere only occurred using N-terminal and C-terminal 6x His-tagged versions of IspS. The protocol for the addition of the 6 His codons at the 5' and 3' ends of the *ispS* gene in the pJF100 Fdii plasmid is given below.

Plasmid pJF100 Fdii producing hexaHistidine tagged Isps (Table 1)

Plasmid pJF100 Fdii was used as template for the independent addition of N- and C-terminal 6x His tag coding sequences to the isoprene synthase (*ispS*) coding region. The 6x His codons were inserted using QuickChange II XL Site Directed Mutagenesis Kit (Agilent Technologies). Each PCR reaction contained 25 ng DNA template and 125 ng each of the forward and reverse primer in 50 µL total volume. Primers (see Supplementary File S1) to insert the N-terminus 6x His tag codons N-Forward and N-Reverse (SEQ ID NOs:56 and 57, respectively) (1). Primers (see Supplementary File S1) to insert the C-terminus 6x His codons were C-Forward and C-Reverse (SEQ ID NOs:58 and 59, respectively) (1). For PCR conditions, *Dpn*I digestion for removal of methylated template DNA and transformation of One Shot Top 10 chemically competent *E. coli* see Beck et al. (1). For each transformation, 150 µL of *E. coli* culture was spread on LB +15 µg chloramphenicol/ml plates and the plates incubated overnight at 30°C. Colonies containing plasmid bearing putative 5' or 3'-terminal *ispS* 6x His codons were used to inoculate 2 ml LB medium each with 15 µg chloramphenicol/mL for overnight growth at 30°C. Plasmid DNA was prepared using a Qiaprep Spin Miniprep (Qiagen). The addition of the 6x His codons was verified by sequencing using primers (see Supplementary File S1) FdxF1 and IDIR2 (SEQ ID NOs:60 and 61, respectively) (1).

Electrocompetent *C. ljungdahlii* cells were transformed with preparations of the pJF100 Fdii IspS N-terminally His-tagged and the pJF100 Fdii IspS C-terminally His-tagged plasmids isolated from Top 10 cells as described earlier. Intactness of the plasmids in the *C. ljungdahlii* transformants was verified as for pJF100 (see also Materials and Methods) by plasmid reisolation, back transformation into *E. coli*, plasmid reisolation and sequencing. Four colonies of the *C. ljungdahlii* transformants, two from each of the two plasmid preps were picked from the enriched MES-F + 5 µg th*i*amphenicol/mL plates and were restreaked on MES-F + 5 µg thiamphenicol/mL plates. These were used to inoculate 60 mL cultures of MES-F + 5 µg thiamphenicol/mL. Cultures of pJF100 Fdii N-term His tagged #1 and #2 and C-term His tagged #1 and #4 were grown on MES-F medium at 37°C to an OD_{600} of 0.806, 0.678 and 0.932 and 0.722, respectively. The cells were pelleted and washed with PBS in preparation for Western blotting as described earlier for the pJF100 transformed *C. ljungdahlii* (also see Materials and Methods). The washed cells were pelleted, frozen in liquid N_2 and stored in a -80°C freezer.

In preparation for Western blotting, the pellets were thawed and resuspended in 1.5, 1.27, 1.75 and 1.35 mL, respectively, of PBS containing 100 µg/mL PMSF. The different volumes were used to assure approximately equal cell densities. The cell suspensions were passaged three times through a French pressure Mini-Cell (SLM-Aminco) at 138 MPa at ~5°C. A portion of the lysates were used immediately for the Western blotting and the remainder aliquoted out, quick frozen in liquid N_2 and stored in a -80°C freezer. Fifteen µL of lysate was mixed with 5 µL of LDS sample buffer (Invitrogen) for each lane of each of three gels: two for Western blots and one stained with Simple Blue SafeStain. Lysates of pJF100 Fdii *C. ljungdahlii* transformants (see Supplementary Figure S6) were used as markers for IspS and Idi.

The electrophoresis gel system was run as described earlier (also see Materials and Methods) and the gels were transferred, incubated with antibody and then with fluorescent-tagged goat anti-rabbit antibody as before (see Supplementary Figure S7).

pJF102 (Table 1)

A plasmid was initially constructed containing mevalonate kinase (*mvk* from *Methanosarcina mazei*), phosphomevalonate kinase (*pmk* from *Saccharomyces cerevisiae*) and mevalonate diphosphate decarboxylase (*mvd* from *Saccharomyces cerevisiae*)) plus *idi* and *ispS*. Placing all of these genes in one plasmid produced no *C. ljungdahlii* transformants following electroporartion, possibly due to the size of the plasmid (9844 bp). Consequently, we divided the genes between two plasmids, one containing *mvk*, *pmk* and *mvd* (pJF102) and the other containing *idi* and *ispS* (pJF101).

Plasmid pJF102 (Supplementary Figure S8, Supplementary File S1) carrying the *mvk*+*pmk*+*mvd* (*mmp*) genes under the control of the P*fdx* promoter with a pCB102 Gram positive replicon and *aad9* spectinomycin resistance marker was constructed as follows. The above-mentioned 9844 bp plasmid containing the *mvk*+*pmk*+*mvd (mmp*) genes was used as a template for PCR amplification of the three-gene operon using primers For+XmaI (Supplementary File S1), and Rev+NheI (Supplementary File S1) yielding a 3.885 kp PCR product (the insert). The PCR product was subsequently gel purified as described previously.

The Heap et al.(3) plasmid pMTL83353 (Supplementary Figure S8) was digested with *Nhe*I and *Xma*I (New England Biolabs) opening the multiple cloning site just downstream of the P*fdx* promoter and RBS. This 4.4 kb vector fragment was gel purified and contains, in addition to the P*fdx* promoter, the *aad9* marker, the pCB102 and ColE1 replicons. The 4.4 kb vector fragment and the 3.885 kb PCR-derived *mmp* insert were ligated with T4 DNA ligase (New

England Biolabs) according to the manufacturer's protocol at room temperature overnight. The ligation was transformed into chemically competent *E. coli* Top10 cells (Invitrogen). After outgrowth in S.O.C. medium, aliquots of the transformation mix were plated onto LB plates with 100 µg spectinomycin/mL and incubated overnight at 30° C.

Transformants were screened by colony PCR with HotStarTaq Master Mix (Qiagen) with primers For-mvk F2 (Supplementary File S1), and Rev-mvdR Supplementary File S1) giving a 1.6 kp PCR product. Correct-sized PCR products from several colonies were sequenced by TempliPhi (GE Health Care Life Sciences) using the same primers, For-mvk F2 (Supplementary File S1), and Primer Rev-mvdR (Supplementary File S1). After confirmation of the correct sequence, the corresponding transformant was grown at 30° C in LB medium containing 100 µg spectinomycin/mL of with shaking at 220 rpm. After overnight growth, the culture was centrifuged at 5,000 × g for 10 minutes and the supernatant decanted. Plasmid DNA was isolated from the pelleted culture using a QIAprep Spin Miniprep Kit (Qiagen).

Electrocompetent *C. ljungdahlii* cells were electroporated with plasmid pJF102 and cultured as described previously (also see Materials and Methods section) except that antibiotic selection was with 1 mg spectinomycin/mL rather than 5 µg thiamphenicol/mL. Confirmation of successful transformation involved plasmid isolation and back transformation into *E. coli* Top10 followed by plasmid reisolation and sequencing as described earlier.

pJF101 (Table 1)

The second vector constructed, pJF101 (P*thl* ii pBP1 *catP*) (Supplementary File S, Supplementary Figure S8), is similar to pJF100 Fdii described above except that the *idi* and *ispS* genes were placed under the control of the promoter and RBS of the thiolase gene (P*thl*) from *C.*

acetobutylicum ATCC824 instead of the ferrodoxin gene (P*fdx*) from *C. sporogenes* NCIMB 10696(3). The P*thl* promoter was derived from the Heap et al. (3) pMTL84422 modular plasmid (Supplementary Figure S8), excised using restriction enzymes *Not*I and *Nde*I and ligated (T4 DNA ligase) into the site in pJF100 Fdii, vacated by the P*fdx* promoter, excised using the same restriction enzymes.

Following transformation and amplification using *E. coli* Top10, this vector was digested with restriction enzymes *Fse*I and *Asc*I (New England Biolabs) to excise the pCB102 replicon. The 4.6 kb vector fragment containing the P*thl* promoter, the *ispS* and *idi* genes, and the *catP* marker was gel purified. The Heap et al. (3) plasmid pMTL82151 was digested with *Fse*I, *Asc*I and *Apa*I (New England Biolabs) and the 2.4 kb insert containing the pBP1 replicon, was gel purified. The pJF100 Fdii (P*thl*)-derived vector fragment and the pMTL82151-derived insert containing the pBP1 replicon were ligated with T4 DNA ligase (New England Biolabs). The 6.9 kb ligation product was transformed into chemically competent *E. coli* Top10 cells (Invitrogen). After outgrowth in S.O.C. medium, aliquots of the transformation mix were plated onto LB plates with 15 µg chloramphenicol/mL and incubated overnight at 30° C.

Transformants were screened by colony PCR with primers pBP1For2 (Supplementary File S1), and pBP1Rev2 (Supplementary File S1) yielding a 2.6 kb PCR product. PCR products from colonies that produced the correct-sized product were sequenced by TempliPhi (GE Health Care Life Sciences) using primers: pMCS337IspSFOR (Supplementary File S1), pMCS337IspSFREV (Supplementary File S1), pMCS337IDIFor (Supplementary File S1), Pmcs337IDIR2 (Supplementary File S1). After sequence confirmation, a transformant was grown at 30° C in LB liquid medium containing 15 µg chloramphenicol/mL with shaking at 220 rpm. After overnight growth, the culture was centrifuged at 5,000 × g for 10 minutes and the

supernatant decanted. Plasmid DNA was isolated from the pelleted culture using a QIAprep Spin Miniprep Kit (Qiagen). The resulting plasmid was named pJF101 (P*thl* ii pBP1 *catP*) (Supplementary Figure S8).

pJF102 + pJF101 double transformant

One of the *C. ljungdahlii* transformants containing plasmid pJF102 (P*fdx* mmp pCB102 *aad9*) was cultured and harvested in the presence of 1 mg spectinomycin/mL under conditions that gave rise to electrocompetent cells (see Materials and Methods section). These were then transformed by electroporation using plasmid pJF101 (P*thl* ii pBP1 *catP*) and selected as described previously (see Materials and Methods section) except that antibiotic selection was with both 1 mg spectinomycin + 5 µg thiamphenicol/mL. Five colonies were picked, streaked on solid MES-F medium containing both antibiotics at the indicated concentrations.
Synthesis of Mvk, PmK, Mvd, Idi and IspS was examined in two of these (#4 and #5), as described previously (see Materials and Methods and Results), by Western blotting (Figure S9) of freshly prepared cell lysates of *C. ljungdahlii* pJF102 + pJF101 double transformants following growth on MES-F + both antibiotics. Only #4 produced all 5 heterologous proteins.

REFERENCES:

1. Beck ZQ, Cervin MA, Chotani GK, Diner BA, Fan J, Peres CM, Sanford KJ, Scotcher MC, Wells DH, Whited GM. 2014. Recombinant anaerobic acetogenic bacteria for production of isoprene and/or industrial bio-products using synthesis gas. US patent US20140234926A1.

2. Beck ZQ, Cervin MA, Chotani GK, Peres CM, Sanford KJ, Scotcher MC, Wells DH, Whited GM. 2013. Fermentation of isoprene and related industrial chemicals using transgenic anaerobic microorganisms. WO Patent WO2013181647A2.
3. Heap JT, Pennington OJ, Cartman ST, Minton NP. 2009. A modular system for Clostridium shuttle plasmids. J Microbiol Methods 78:79-85.
4. Keasling JD, Newman JD, Pitera DJ**.** 2006. Preparation of plasmid vectors encoding mevalonate pathway enzymes for enhancing production of isoprenoid compounds in transgenic host cells. US patent US20060079476A1.

Figure S3

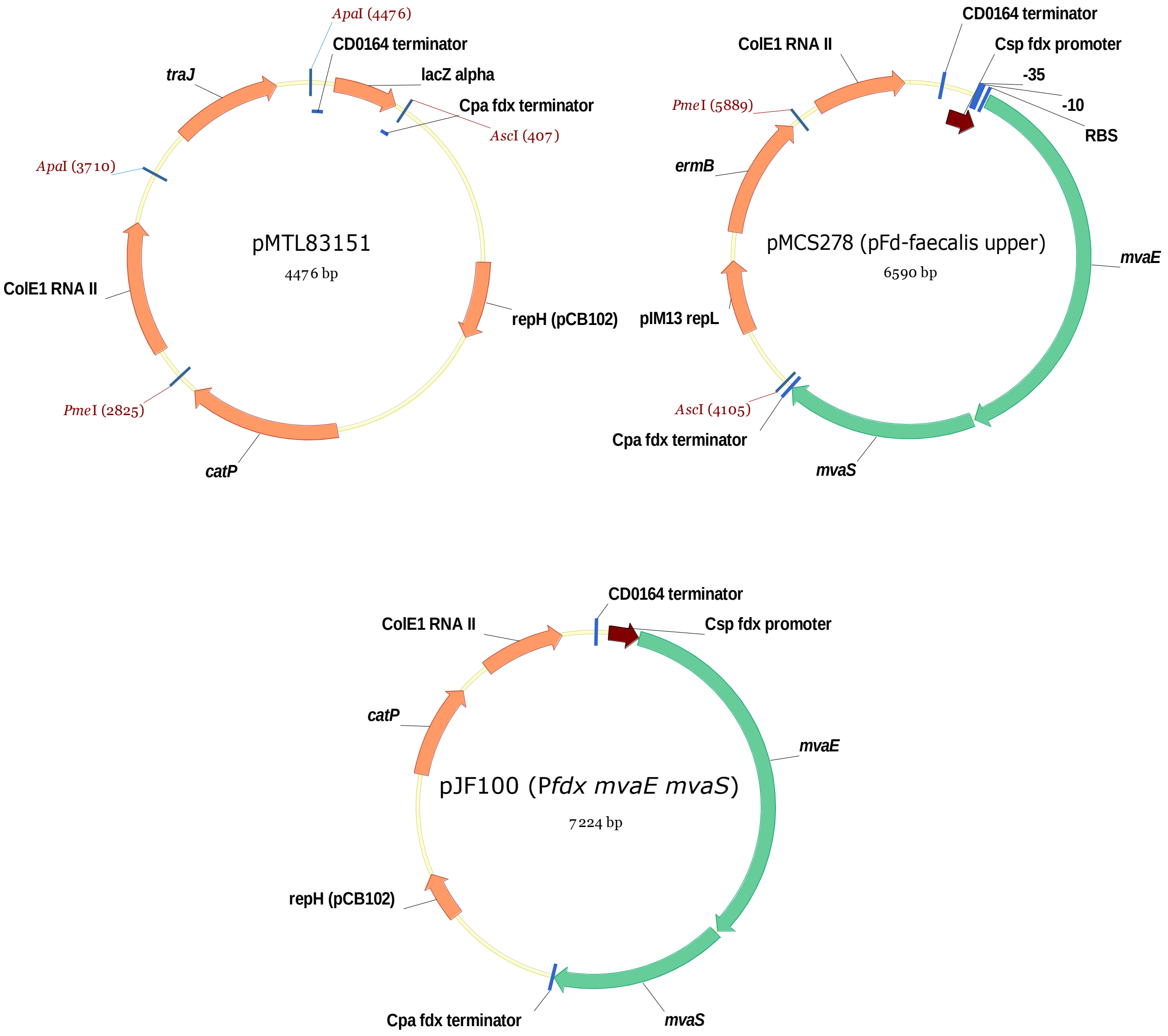

Figure S3, legend - Plasmid maps for the *Clostridium-E. coli* shuttle plasmids pMCS278, pMTL83151 and pJF100 (Table 1).

Figure S4

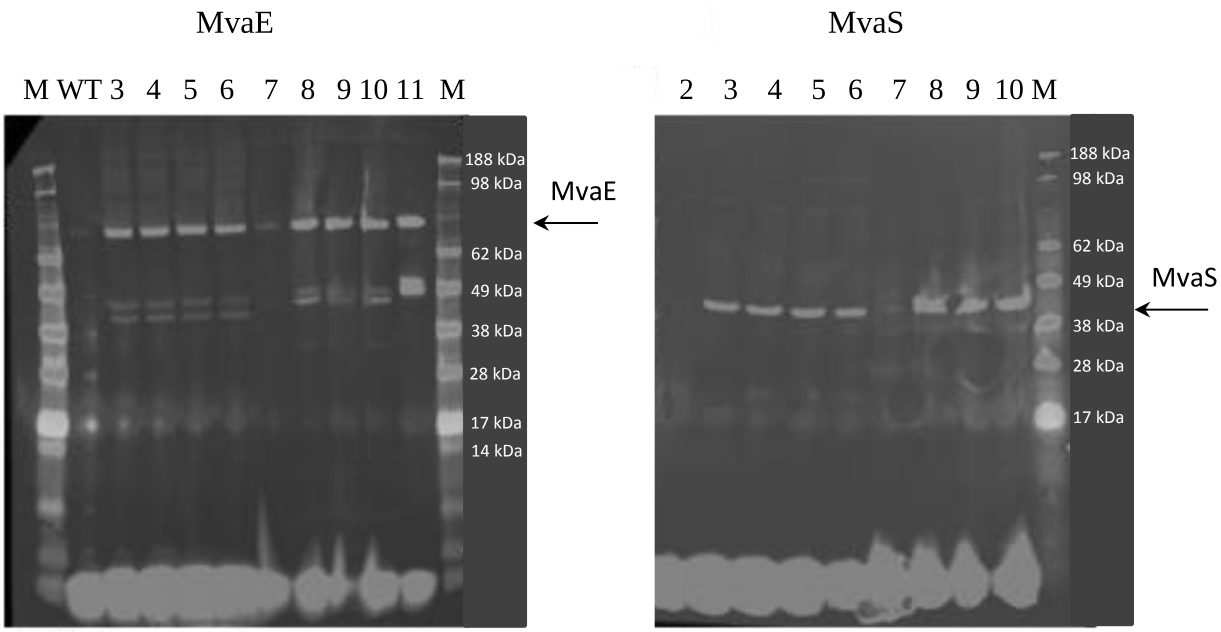

M: Protein MW Markers
WT: WT *C. ljungdahlii*
3-6: pJF100 in *C. ljungdahlii* (#2,4,6,8)
7: pMTL83151 in *E. coli* TV3007
8-10: pJF100 (#2,6,8) in *E. coli* TV3007
11: MvE purified protein – see scale

2: WT *C. ljungdahlii*
3-6: pJF100 in *C. ljungdahlii* (#2,4,6,8)
7: pMTL83151 in *E. coli* TV3007
8-10: pJF100 (#2,6,8) in *E. coli* TV3007
M: Protein MW Markers – see scale

Figure S4 - Western blots of whole cell lysates of *C. ljungdahlii* WT and the pJF100 transformant and *E. coli* TV3007 pMTL83151 and pJF100 transformants, showing the synthesis of MvaE and MvaS (see Materials and Methods) only in the presence of pJF100. Lanes: lane M. protein molecular weight markers; lane WT. *C. ljungdahlii* WT (wild type); lanes 3-6. pJF100 in *C. ljungdahlii* Nos. 2, 4, 6 and 8, respectively; lane 7. pMTL83151 in *E. coli* TV3007; lanes 8-10 pJF100 Nos. 2, 6 and 8, respectively in E. coli TV3007; lane 11. MvaE purified protein. (Left) blot probed with anti-MvaE antiserum diluted 1:1000 in Blocking solution (Invitrogen); (Right) blot probed with anti-MvaS antiserum diluted 1:1000 in Blocking solution (Invitrogen). The secondary antibody solution was 2 µg Alexa Fluor 488 goat anti-rabbit IgG (H+L)/mL. The calculated molecular mases of MvaE and MvaS are 86.5 and 42.1 kDa, respectively.

Figure S5

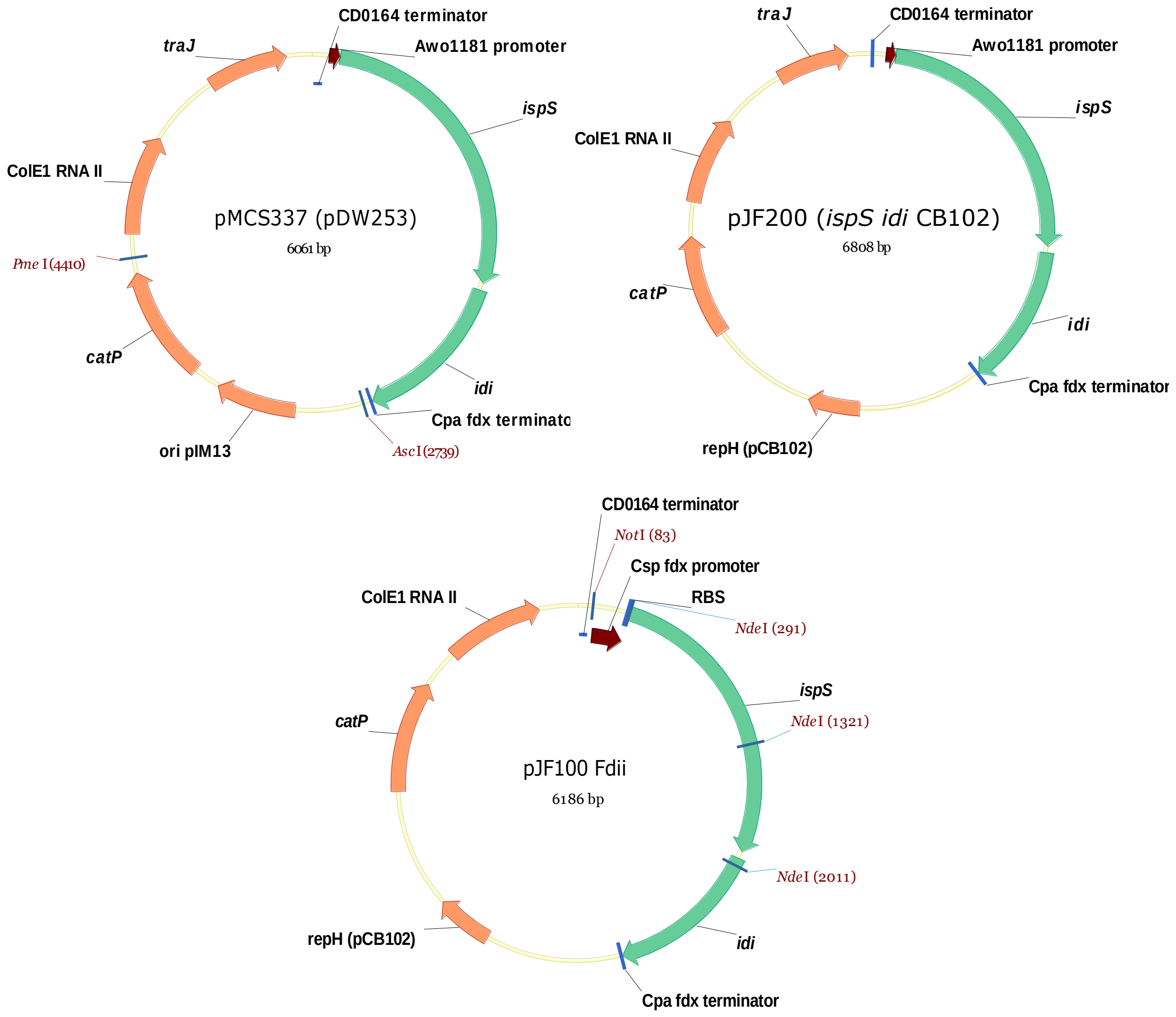

Figure S5, legend - Plasmid maps for the *Clostridium-E. coli* shuttle plasmids pMCS337 (pDW253), pJF200 and pJF100 Fdii (Table 1).

Figure S6

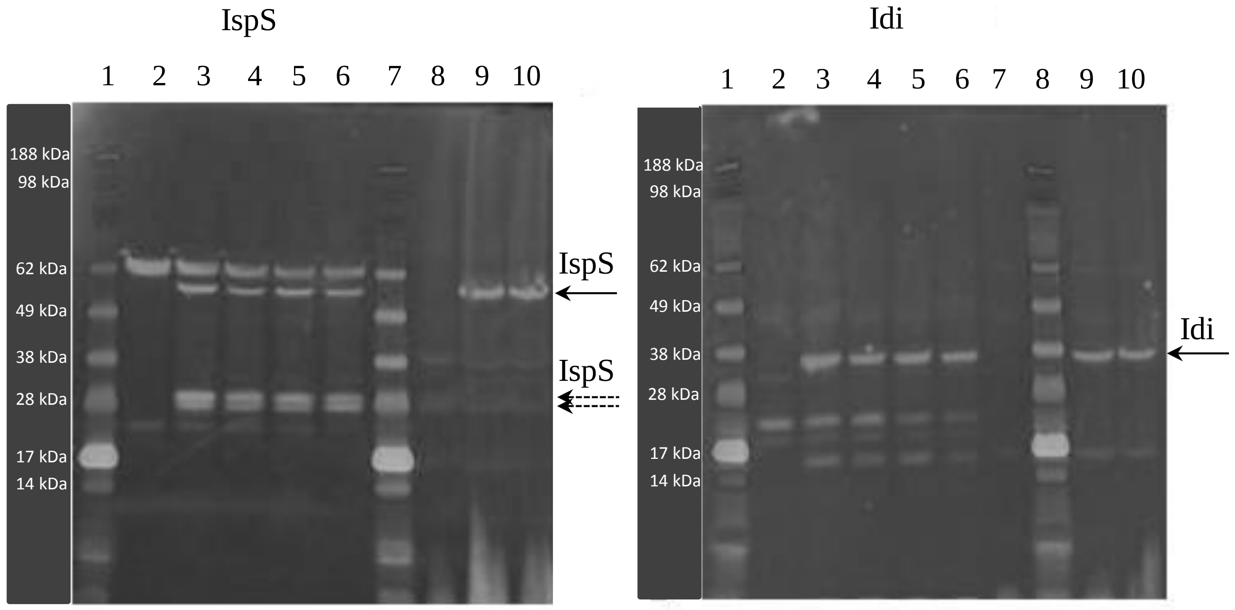

1: Protein MW markers – see scale
2: WT *C. ljungdahlii*
3-6: pJF100 Fdii in *C. ljungdahlii*
7: Protein MW Marker
8: pMTL83151 in *E. coli* TV3007
9-10: pJF200 nos. 1LD and 2LD in *E. coli* TV3007

1: Protein MW markers – see scale
2: WT *C. ljungdahlii*
3-6: pJF100 Fdii in *C. ljungdahlii*
7: pMTL83151 in *E. coli* TV3007
8: Protein MW Marker
9-10: pJF200 nos.1LD and 2LD in *E. coli* TV3007

Figure S6 - Western blots of whole cell lysates of *C. ljungdahlii* WT and pJF 100 Fdii transformant and *E. coli* TV3007 pMTL83151 and pJF200 transformants showing the synthesis of Idi and IspS (see Materials and Methods). (Left) Lanes: lanes 1 and 7. (protein molecular weight markers); lane 2. *C. ljungdahlii* WT (wild type); lanes 3-6. pJF100 Fdii in *C. ljungdahlii* four different transformants; lane 8. pMTL83151 in *E. coli* TV3007; lanes 9 and 10. pJF200 in *E. coli* nos. 1LD and 2LD, respectively. Blot probed with anti-IspS antiserum diluted 1:1000 in Blocking solution (Invitrogen); (Right) Lanes: lanes 1 and 8. protein molecular weight markers; lane 2. *C. ljungdahlii* WT (wild type); lanes 3-6. pJF100 Fdii in *C. ljungdahlii* four different transformants; lane 7. pMTL83151 in *E. coli* TV3007; lanes 9 and 10. pJF200 in *E. coli* nos.

1LD and 2LD. Blot probed with anti-Idi antiserum diluted 1:1000 in Blocking solution (Invitrogen). The secondary antibody solution was 2 µg Alexa Fluor 488 goat anti-rabbit IgG (H+L)/mL. The calculated molecular masses for IspS and Idi are 62.6 and 33.4 kDa, respectively. Partial proteolysis fragments of IspS are observed at 27-8 kDa.

Figure S7

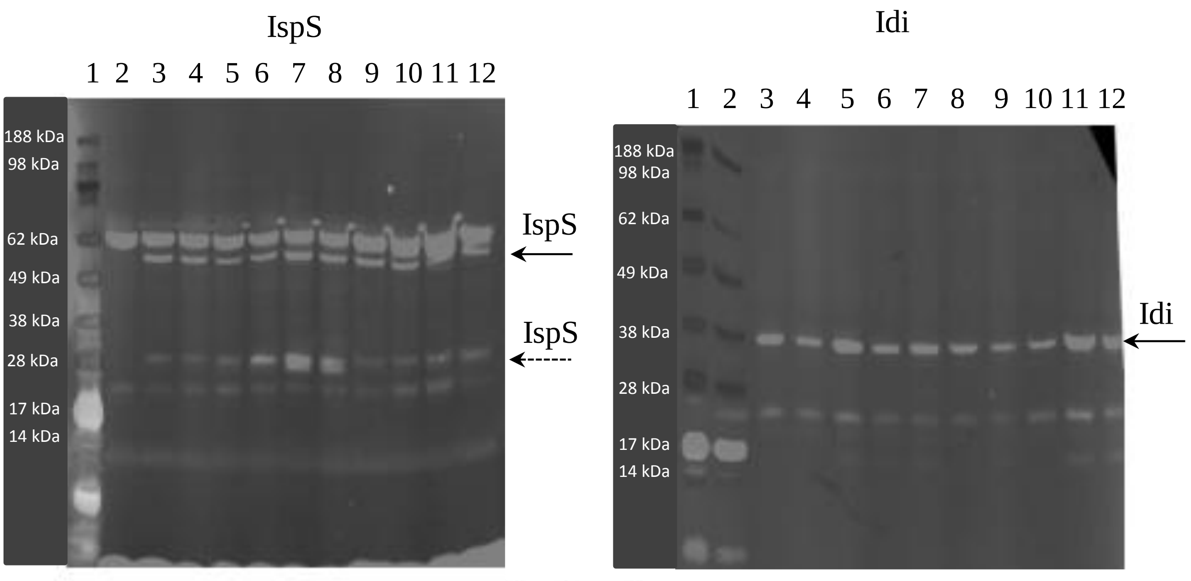

1: Protein MW markers – see scale
2: WT *C. ljungdahlii (C.l.)*
3-4: pJF100 Fdii with N-term His IspS in *C.l.*
5-6: pJF100 Fdii with C-term His IspS in *C.l.*
7-8: pJF100 Fdii in *C.l.*
9-10: 1.5x pJF100 Fdii with N-term His IspS in *C.l.*
11-12: 1.5x pJF100 Fdii with C-term His IspS in *C.l.*

1: Protein MW markers – see scale
2: WT *C. ljungdahlii (C.l.)*
3-4: pJF100 Fdii with N-term His IspS in *C.l.*
5-6: pJF100 Fdii with C-term His IspS in *C.l.*
7-8: pJF100 Fdii in *C.l.*
9-10: 1.5x pJF100 Fdii with N-term His IspS in *C.l.*
11-12: 1.5x pJF100 Fdii with C-term His IspS in *C.l.*

Figure S7- Western blots of whole cell lysates of *C. ljungdahlii* WT and pJF 100 Fdii N-terminal and C-terminal IspS His-tagged transformants showing the synthesis of Idi and IspS (N-terminal and C-terminal 6xHis-tagged). Lanes: lane 1. protein molecular weight markers; lane 2. *C. ljungdahlii* WT (wild type); lanes 3-4. pJF100 Fdii with N-terminal IspS His-tag in *C.*

ljungdahlii; lanes 5-6. pJF100 Fdii with C-terminal IspS His-tag in *C. ljungdahlii*); lanes 7-8. pJF100 Fdii without His-tag; lanes 9-10. 1.5x pJF100 Fdii with N-terminal IspS His-tag in *C. ljungdahlii*; lanes 11-12. 1.5x pJF100 Fdii with C-terminal IspS His –tag in *C. ljungdahlii*. (Left) Blot probed with anti-IspS antiserum diluted 1:1000 in Blocking solution (Invitrogen); (Right) Blot probed with anti-Idi antiserum diluted 1:1000 in Blocking solution (Invitrogen). The secondary antibody solution was 2 μg Alexa Fluor 488 goat anti-rabbit IgG (H+L)/mL. The calculated molecular masses for IspS and Idi are 63.5 and 33.4 kDa, respectively. Partial proteolysis fragments of IspS are observed at 27-9 kDa.

Figure S8

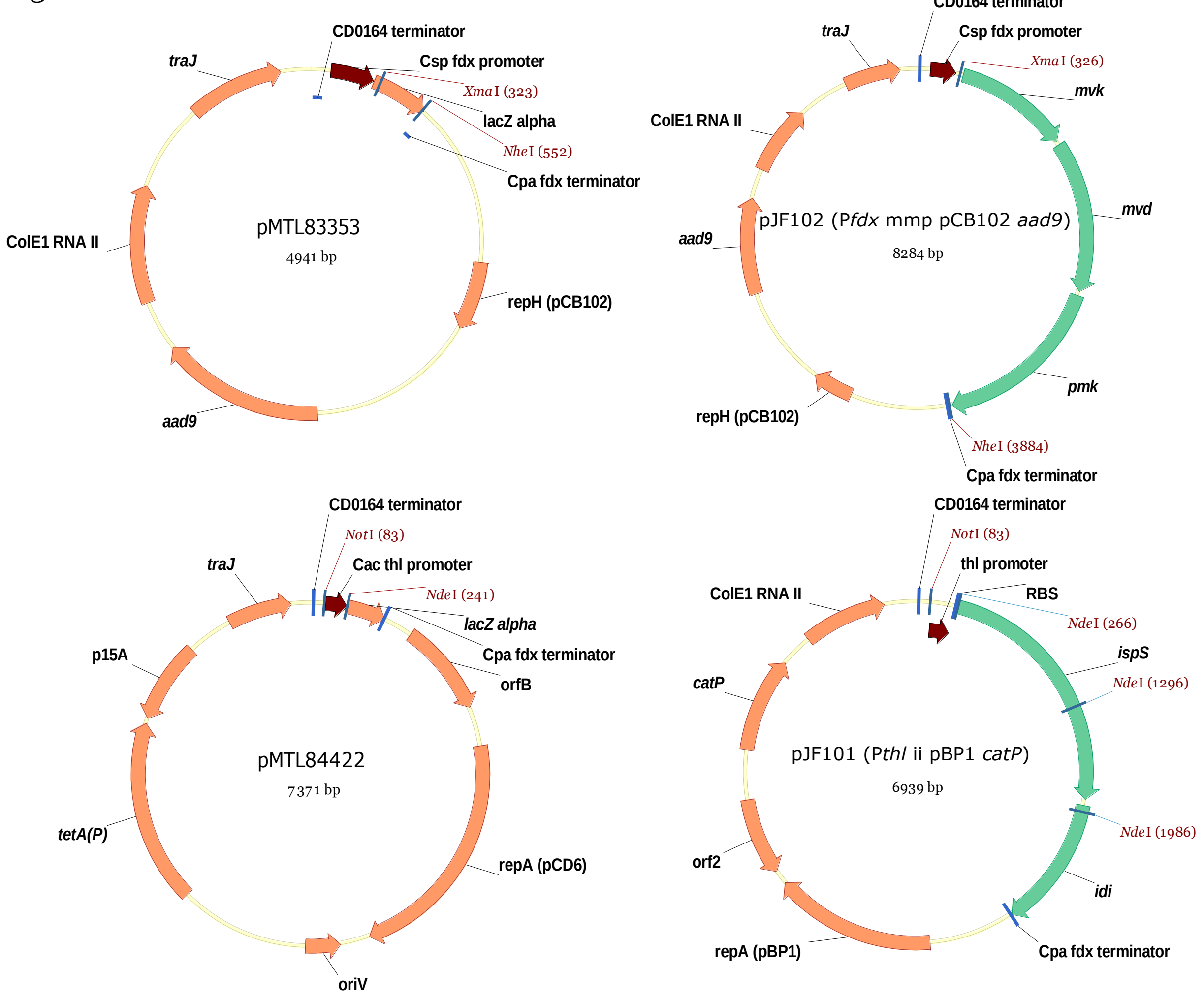

Figure S8, legend - Plasmid maps for the *Clostridium-E. coli* shuttle plasmids pMTL83353, pMTL84422, and pJF102 and pJF101 (see Table 1 and Supplementary File S1).

Figure S9

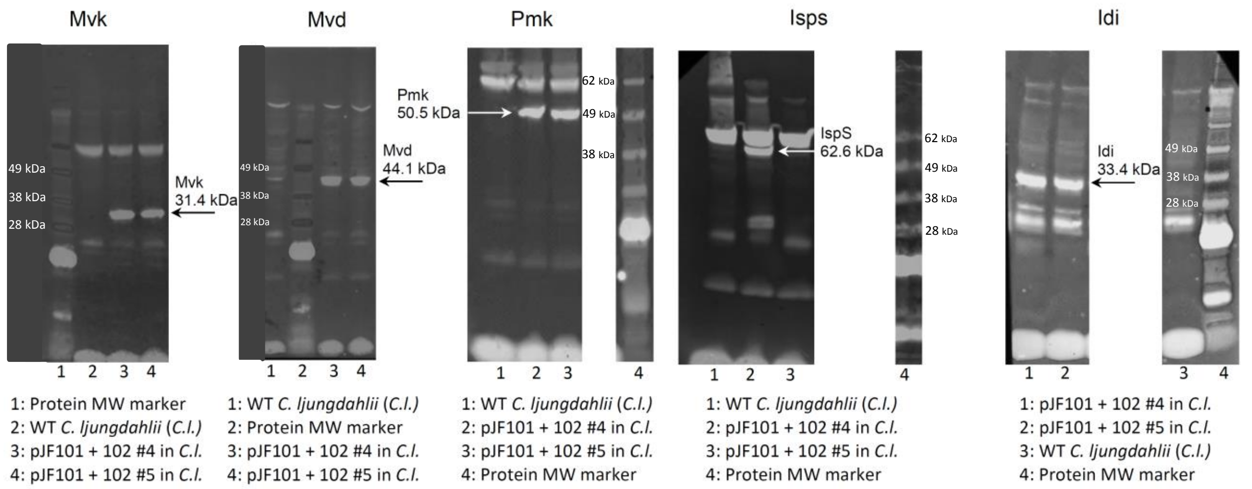

Figure S9 – Western blots of cell lysates of the doubly transformed *C. ljungdahlii* strains (#4 and #5) transformed with pJF101+pJF102 showing the synthesis of Mvk, Mvd, Pmk, IspS and Idi. For anti-Pmk and anti-IspS, Lanes 1-4: WT *C. ljungdahlii*, pJF101+pJF102 transformants #4 and #5, protein molecular weight markers; for anti-Mvk, lanes 1-4: protein molecular weight markers, WT *C. ljungdahlii*, pJF101+pJF102 transformants #4 and #5; for anti-Mvd, lanes 1-4: WT *C. ljungdahlii*, protein molecular weight markers, pJF101+pJF102 transformants #4 and #5; for anti-Idi, lanes 1-4: pJF101+pJF102 #4 and #5, WT *C. ljungdahlii*, protein molecular weight markers. The antisera for IspS and Idi were diluted 1:1000 in Blocking solution. The antisera for Mvk, Mvd and Pmk were diluted 1:2000 in Blocking solution. The secondary antibody solution was 2 µg Alexa Fluor 488 goat anti-rabbit IgG (H+L)/mL. The calculated molecular masses for Mvk, Mvd, Pmk, IspS and Idi are 31.4, 44.1, 50.5, 62.6 and 33.4 kDa, respectively.
